# Nanocarriers based therapy and diagnosis of brain diseases: cross the blood-brain barrier

**DOI:** 10.1080/14686996.2025.2554048

**Published:** 2025-09-09

**Authors:** Lijun An, Jinwei Zhang, Xiaobo Wang, Yuanyuan Ge, Kunhui Sun, Junlin Dong, Ping Wang, Wei Li, Meifang Li, Xuelei Hu, Bing Wang, Xie-An Yu

**Affiliations:** a NMPA Key Laboratory for Bioequivalence Research of Generic Drug Evaluation, NMPA Key Laboratory for Quality Research and Evaluation of Traditional Chinese Medicine, Shenzhen Institute for Drug Control, Shenzhen, China; b Key Laboratory for Green Chemical Engineering Process of Ministry of Education, Hubei key Laboratory of Novel Reactor and Green Chemical Technology, School of Chemical Engineering and Pharmacy, Wuhan Institute of Technology, Wuhan, China; c Chongqing University, Chongqing, China

**Keywords:** Blood-brain barrier, nanocarriers, brain disease, treatment, diagnosis

## Abstract

The blood-brain barrier (BBB) is the protective interface that isolates the central nervous system from circulating blood, which restricts approximately 98% of small molecule drugs and nearly all large molecules from entering the brain. Current methods to bypass the BBB, such as laser-guided interstitial thermal therapy and magnetic resonance guided focused ultrasound, are fraught with risks like impairing BBB integrity and brain damage, and are not suitable for long-term treatment. Nanocarriers have emerged as promising tools due to their ability to enhance drug delivery across the BBB while minimizing systemic toxicity. These nanocarriers leverage mechanisms including receptor-mediated, carrier-mediated, cell mediated and extra-stimuli mediated transport to improve BBB traverse and brain targeting. The review evaluates these strategies separately, discussing their potential and limitations for clinical application, and highlights recent advancements in integrating and optimizing nanocarriers utilizing synergistic strategies for the treatment and diagnosis of neurological disorders, including tumors, Alzheimer’s disease, Parkinson’s disease, and brain infections.

## IMPACT STATEMENT

BBB represents a formidable challenge in brain diseases. The review evaluates nanocarriers strategies across BBB, discussing potential and limitations for clinical application, highlights advancements for the theranostics of brain diseases.

## GRAPHICAL ABSTRACT

**Figure:**
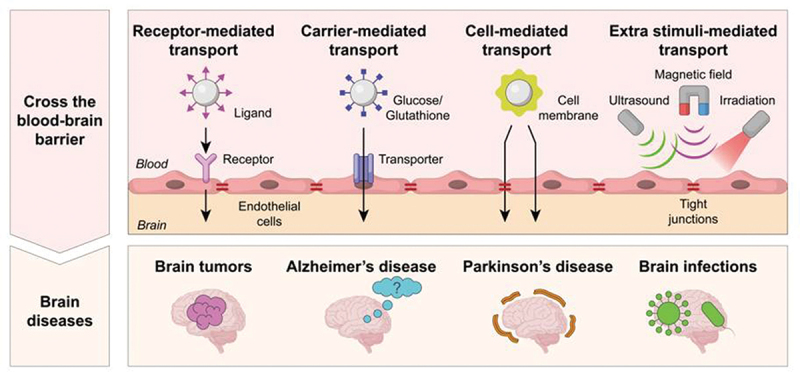

## 1. Introduction

The blood-brain barrier (BBB) is a highly selective and tightly regulated endothelial interface that limits the entry of therapeutic agents into the brain, posing significant challenges for the treatment of neurological disorders. It was reported that approximately 98% of small molecule drugs (trastuzumab, doxorubicin, vincristine, etc.) and nearly all large molecules (recombinant proteins, antibodies, nucleic acids, etc.) are regularly blocked from entering the brain [1,2]. Currently, for therapeutics that manage to cross the BBB, several significant clinical challenges arise. Systemic cytotoxicity is a primary concern, as the drugs often lack selectivity, causing unintended harm to non-target tissues [3]. Additionally, the methods used to facilitate drug entry into the brain may further disrupt the BBB, complicating treatment. Furthermore, those drugs that do reach the brain often show poor penetration, with most central nervous system drugs achieving only a 1–4% penetration rate due to low BBB permeability and rapid in vivo clearance [4,5]. Alternative approaches, such as bypassing the BBB through direct drug delivery to the brain via invasive procedures like intracerebral injections or intrathecal administration, have been explored. These methods, while capable of achieving higher local drug concentrations, are often associated with significant risks, including infection, bleeding, and damage to the brain tissue, making them less appealing for routine clinical use [6,7]. Moreover, these invasive techniques are economically costly and may not be feasible for chronic conditions requiring long-term treatment [8]. Therefore, there is a pressing need for safer, more effective strategies that can enhance drug delivery to the brain while minimizing systemic exposure and side effects.

Nanocarriers have garnered significant attention in therapeutic or diagnostic agents’ delivery research, thanks to their numerous advantages. These include a higher capacity for agent loading, reduced dosage and frequency of administration, and favorable biocompatibility which results in fewer side effects [9,10]. Furthermore, they offer enhanced stability, bioavailability, and biodegradability. They also contribute to decreased acquired drug resistance, minimal toxicity and immunogenicity, reduced off-target effects, and compatibility with various administration routes such as intranasal, oral, intravenous, and intramuscular [11,12]. Additionally, they enable sustained and controlled drug release, possess unique physical, electronic, chemical, and optical properties, and allow for efficient tracking of agents’ delivery [13,14]. Nanocarriers also exhibited their superiority in BBB penetration and brain targeting via using less invasive theragnostic methods, via mechanisms including receptor-mediated transport (RMT), carrier-mediated transport, cell-mediated transport (CMT) and extra stimuli-mediated transport (ESMT). These nanocarriers are thus being developed as safe, effective, and practical tools for diagnosing and treating central nervous system disorders, with some having entered clinical trials (Table 1).

**Table 1.** Nanocarriers in clinical trials for brain diseases.

Diseases	Type of Nanocarriers	Treatment	Conditions	Trials ID and Study Phase	Current situation	References
Brain tumor	Iron oxide nanoparticles	NanoTherm therapy	Glioblastoma multiforme	NCT06271421 Not Applicable (NA)	Recruiting	[15]
	Spherical nucleic acid gold nanoparticle (NU-0129)	Targeted molecular therapy	Gliosarcoma Recurrent Glioblastoma	NCT03020017 Early Phase 1	Completed	[16]
	EGFR (Vectibix® Sequence)-targeted EnGeneIC dream vectors containing doxorubicin (EGFR(V)-EDV-Dox)	NA	Glioblastoma Astrocytoma, Grade IV	NCT02766699 Phase 1	Unknown	[17]
	Panobinostat nanoparticle (MTX110)	Convection-Enhanced Delivery	Diffuse Intrinsic Pontine Glioma	NCT03566199 Phase 1/2	Posted	[18]
	Polysiloxane Gd-Chelates based nanoparticles (AGulX)	Whole Brain Radiation Therapy	Brain Metastases	NCT02820454 Phase 1	Published	[19,20]
		In combination with radiotherapy and Temozolomide	Glioblastoma	NCT04881032 Phase 1/2	Published	[21,22]
		Whole Brain Radiation Therapy	Brain Metastases, Adult Radiotherapy	NCT03818386 Phase 2	Active, not recruiting	[23]
		Stereotactic Radiation	Brain Metastases	NCT04094077 Phase 2	Terminated	[24]
		Stereotactic Radiation	Brain Cancer Brain Metastases	NCT04899908 Phase 2	Recruiting	[25]
AD	Intranasal Nanoparticles of APH-1105	NA	Dementia AD	NCT03806478 Phase 2	Not yet recruiting	[26]
PD	Gold Nanocrystals (CNM-Au8)	Magnetic Resonance Spectroscopy (31P-MRS)	PD	NCT03815916 Phase 2	Posted	[27]
Stroke	Iron nanoparticles	Magnetically enhanced diffusion	Ischemic Stroke Stroke	NCT06495671 NA	Not yet recruiting	[28]
			Acute Ischemic Stroke Cerebral Arterial Disease	NCT06052969 NA	Recruiting	[29]

**Figure 1.**
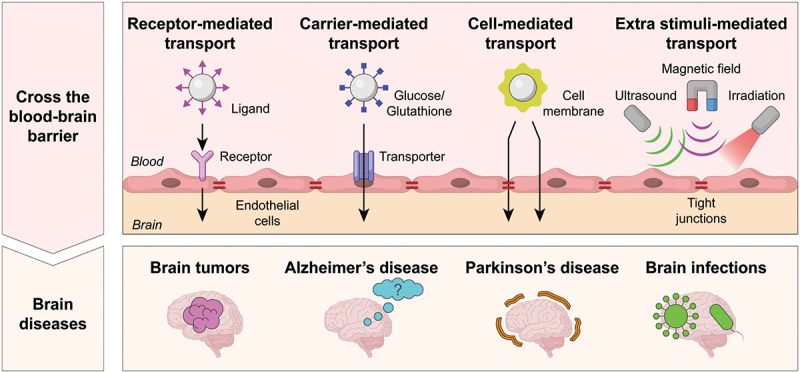
Scheme of the mechanisms of nanocarriers to cross the BBB, and the brain diseases.

Herein, we provide a detailed overview of the structure and function of the BBB, laying the foundation for understanding the challenges associated with brain drug delivery. We then delve into the various mechanisms by which nanocarriers can be designed to cross the BBB, including RMT, carrier-mediated transport, CMT, and ESMT. Each of these strategies is critically evaluated, highlighting their advantages, limitations, and potential for clinical translation separately. Finally, we discuss recent advancements in the use of integrated and synergistic strategies to enhance nanocarrier-mediated BBB penetration for the treatment and diagnosis of brain diseases, including tumors, Alzheimer’s disease (AD), Parkinson’s disease (PD), and infections (Figure 1). Through this review, we aim to provide insights and guidance for the future development of nanocarrier systems designed to effectively traverse the BBB and improve therapeutic outcomes in neurological disorders.

## 2. Structure of the blood-brain barrier

The blood-brain barrier (BBB) is the protective interface that isolates the central nervous system from circulating blood. Its close proximity to the brain parenchyma offers a viable opportunity for drug delivery in treating brain diseases [1,4]. Therefore, understanding the structure of the BBB is crucial for the effective delivery of nanoparticles.

### 2.1. Physiological structure of the blood-brain barrier

**Figure 2.**
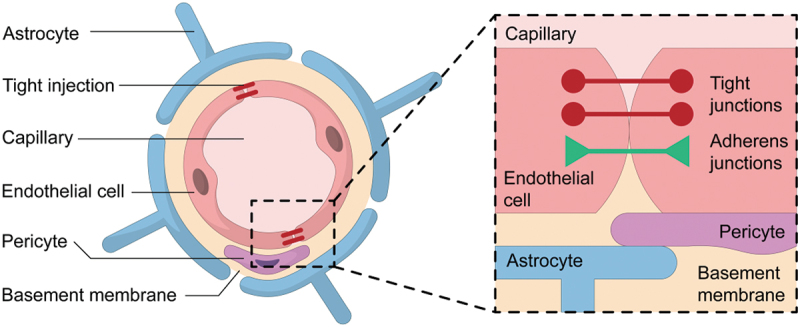
Structure of the BBB.

Generally, the BBB is composed of endothelial cells, pericytes, basement membrane and astrocytes. A single layer of endothelial cells from the blood vessel wall, which is tightly enveloped by pericytes. These cells are further embedded in the basement membrane and then wrapped by astrocytic end-feet [5,15] (Figure 2).

#### 2.1.1. Endothelial cells

Endothelial cells are considered as the core anatomical structure of the BBB, for forming the largest interface for blood-brain exchange. Endothelial cells in the BBB are unique due to the barrier performance, compared to those in peripheral tissues [31,32]. Firstly, without endothelial fenestrations, endothelial cells are fastened as the highly selective barrier via tight junctions, composed of specific proteins with claudins, occludins, and junctional adhesion molecules included [33–35]. Tight junctions restrict the effective pore size between endothelial cells as 1.4–1.8 nm [36], and assistant to prevent most water-soluble substances, ions, and pathogens from paracellular transport through the passing between endothelial cells [37], thereby protecting the brain from harmful substances. Secondly, they exhibit low level of transcytosis to restrict large molecules such as proteins and lipids from crossing into the brain tissue [38,39]. This characteristic further reinforces the selective permeability of the BBB. But they also possess selective transport mechanisms, such as carrier-mediated transport represented by glucose transporters, and receptor-mediated transport exemplified by insulin transport [40–42], to ensure that the brain receives essential nutrients and signaling molecules. At last, they prevent excessive infiltration of immune cells such as peripheral leukocytes via expressing low levels of cell adhesion molecules, thus reducing the inflammatory responses and protecting nervous tissues from inflammatory damage [43–45].

#### 2.1.2. Pericytes

Pericytes closely wrap the capillary blood vessels of the BBB, working in conjunction with endothelial cells and basement membrane that provides structural support and maintain capillary stability and barrier integrity [46,47]. They also play a critical role in BBB formation, particularly promoting the formation and maintenance of tight junctions between endothelial cells, via secreting growth factors and signaling molecules, such as platelet-derived growth factor-B (PDGF-B) and transforming growth factor-β (TGF-β) [48–50]. Furthermore, pericytes can limit the infiltration of peripheral immune cells, regulate vascular growth and development as well as blood flow. They also participate in the repair process of the BBB by migration, proliferation, and differentiation [51].

#### 2.1.3. Basement membrane

Basement membrane is a complex extracellular matrix structure collaboratively secreted by endothelial cells and pericytes, which is composed with various extracellular matrix proteins, including collagen, laminin, perlecan, and fibronectin [52–56]. Because of these compositions, the basement membrane is regarded as a physical barrier with dense network structure, which restricts the passage of large molecules, pathogens, and toxins to brain [57]. It also participates in signaling between endothelial cells and pericytes, and regulates their differentiation, migration, and proliferation [58].

#### 2.1.4. Astrocytes

Astrocytes are a type of glial cell that encase blood vessels along with pericytes, functioning as a critical interface between neurons and endothelial cells [59]. They preserve the structural and functional integrity of the BBB, due to these interact with endothelial cells and basement membrane via their end-feet [60]. They can also modulate behavior endothelial cells and pericytes by secreting signaling molecules, such as cytokines and growth factors [61].

### 2.2. The blood-brain barrier under pathological conditions

#### 2.2.1. Brain tumors

The blood-brain tumor barrier (BBTB) exhibits unique structural and functional changes compared to a healthy BBB. In brain tumors such as glioblastoma and medulloblastoma, the BBTB features disrupted microvascular networks with newly formed, leaky vessels primarily in the tumor core, while the peripheral regions of the tumor retain a more intact barrier [62,63]. This results in a heterogeneous and disorganized vasculature, characterized by increased fenestrations and intercellular gaps, leading to enhanced paracellular transport of nanocarriers [64].

#### 2.2.2. Alzheimer’s disease

AD is a leading cause of dementia, marked by the accumulation of amyloid-β (Aβ) plaques and tau protein deposits. These pathologies contribute to neurovascular unit disruption, including BBB breakdown, pericyte death, and altered astrocyte morphology [65,66]. Additionally, endothelial and mitochondrial abnormalities, tight junction disturbances, and reactive gliosis further compromise BBB integrity [67]. The resulting vascular and cellular changes exacerbate AD progression, leading to cognitive decline, but also provide opportunities for nanocarrier-based therapy and diagnosis.

#### 2.2.3. Parkinson’s disease

PD is a progressive neurodegenerative disorder characterized by the loss of dopaminergic neurons in the substantia nigra pars compacta and the formation of Lewy bodies made of insoluble α-synuclein [68]. In PD, early-stage angiogenesis increases vessel density, while late-stage disease sees reduced vessel density and significant BBB disruption. Histological analyses reveal capillary leakage, extravasated erythrocytes, and protein accumulation, with activated microglia around blood vessels [69]. BBB integrity is compromised, as indicated by elevated cerebrospinal fluid/serum ratios of albumin and IgG [70]. PD manifests with motor symptoms like tremors, rigidity, and bradykinesia, and nonmotor symptoms such as dementia and depression. BBB dysfunction exacerbates dopaminergic neuron loss, impairing motor function and coordination [71].

#### 2.2.4. Brain infections

Brain infections are a group of diseases caused by pathogens like bacteria, viruses, fungi, or parasites. Conditions such as meningitis, encephalitis, and brain abscesses fall within this category and are characterized by symptoms including fever, headache, altered consciousness, and seizures. Given the critical nature of central nervous system, these infections often present high morbidity and mortality rates [72]. During such infections, the inflammatory response may compromise the BBB, increasing its permeability. This change allows pathogens and immune cells to infiltrate brain tissue more easily, worsening the infection and inflammation [45].

#### 2.2.5. Stroke

Stroke is a severe cerebrovascular condition with two main types: ischemic and hemorrhagic. Ischemic strokes, accounting for 80–87% of cases, result from vessel blockage, causing a cascade of ischemic damage, including neuronal death and neuroinflammation [73]. The BBB undergoes complex changes across stroke stages: in the hyperacute phase, hypoxia leads to BBB disruption and cytotoxic edema; in the acute phase, inflammation further compromises the BBB, increasing permeability and hemorrhagic risk; in the subacute phase, repair mechanisms like angiogenesis are activated; and in the chronic phase, the BBB is partially restored through increased expression of tight junction proteins [74]. Overall, BBB dysfunction in stroke involves endothelial damage, tight junction degradation, and altered neuroinflammatory responses, impacting drug delivery and brain recovery. Additionally, some nanocarriers are specifically designed for repairing the BBB in stroke [75,76].

## 3. Strategies of nanocarriers to cross the BBB

In the context of nanocarrier delivery, there are four main pathways to cross the BBB and enter the central nervous system, including receptor-mediated transport (RMT), carrier-mediated transport, cell-mediated transport (CMT) and extra stimuli-mediated transport (ESMT). Their mechanisms have been briefly described below, correspondingly.

### 3.1. Receptor-mediated transport

RMT has garnered significant interest among the various strategies for nanocarriers to traverse the BBB. It typically refers to the process by which ligands modified on nanocarriers cross the BBB through specific binding to receptors on the endothelial cells of the BBB.

Initially, these ligands bind to their corresponding receptors, such as transferrin receptors (TfR), low-density lipoprotein receptors, or diphtheria toxin receptors, on the luminal surface of endothelial cells at the BBB [77]. Following receptor-ligand binding, the nanocarriers are endocytosed into the endothelial cells within endocytic vesicles, primarily via clathrin- or caveolin-mediated pathways [78,79]. These vesicles are then sorted and transported; some are sent to lysosomes for degradation, while others are directed to the basolateral membrane of the endothelial cells [78]. Ultimately, these vesicles fuse with the abluminal membrane of the endothelial cells and release their contents, the nanocarriers, into the brain tissue. Throughout this process, receptors may be recycled back to the endothelial cell membrane to participate in new transport cycles [80].

It is noteworthy that RMT is characterized by two key features: high specificity and efficiency. Its specificity lies in its ability to recognize and transport nanocarriers modified with specific ligands, while its efficiency is derived from the utilization of the natural transport mechanisms of endothelial cells, allowing nanocarriers to cross the BBB without compromising the barrier’s integrity. In the following sections, examples are categorized and introduced according to different types of ligands, including proteins, peptides, antibodies, aptamers, and other small molecules.

#### 3.1.1. Proteins

Generally, nanocarriers modified with proteins were expected to mimic the natural behavior of endogenous proteins for successful penetration of the BBB, via recognizing and interacting with their corresponding receptors [12]. These endogenous proteins such as lactoferrin (Lf), transferrin (Tf), and apolipoproteins (Apo) are usually taken into consideration.

For instance, Lf modification on nanocarriers can target overexpressed receptors on the BBB, enhancing nanoparticle attachment to endothelial cells with improved chemotherapeutic drug delivery across the BBB [81]. Wang et al. developed albumin and lactoferrin-based nanoparticles that effectively crossed the BBB by targeting low-density lipoprotein receptor-related protein-1 (LRP-1), which is overexpressed in both BBB endothelial cells and glioma cells. In a bEnd.3 cell monolayer model, these dual-protein nanoparticles demonstrated 4.5 times greater BBB penetration compared to albumin-only nanoparticles. Additionally, in an orthotopic glioma mouse model, the dual-protein nanoparticles showed a fivefold increase in brain accumulation, confirming their enhanced BBB penetration and targeted delivery to glioma cell [82]. In other cases, Lf-modified nanodots were engineered to enhance the BBB permeability for Parkinson’s disease therapy. In an endothelial cell monolayer model, these nanodots achieved 20% penetration to the basolateral side, versus 7% for non-modified counterparts, highlighting the role of Lf in facilitating BBB crossing. A blocking experiment with free Lf reduced penetration to 8.5%, underscoring the essential function of Lf in the BBB traversal [83].

Lf coating also helps for nanocarrier based target imaging of brain diseases. Recently, Ge et al. prepared Lf-conjugated nanocrystals enhance BBB permeability and glioma targeting through Lf modification. In transwell model, approximately 30% of Lf-conjugated nanocrystals crossed the BBB, compared to 10% for non-conjugated ones. In vivo, these nanocrystals accumulated in glioma after 1 h, with peak fluorescence at 8 h, demonstrating effective BBB penetration and targeted glioma imaging. In contrast, non-conjugated nanocrystals showed no significant brain fluorescence [84]. Similarly, Lf modified carbon dots with red fluorescence can cross the BBB via Lf receptor. Experiments in PD mice showed selective accumulation in dopaminergic neurons, effectively reducing oxidative stress and improving behavior with PD imaging, confirming successful BBB penetration [85].

**Figure 3.**
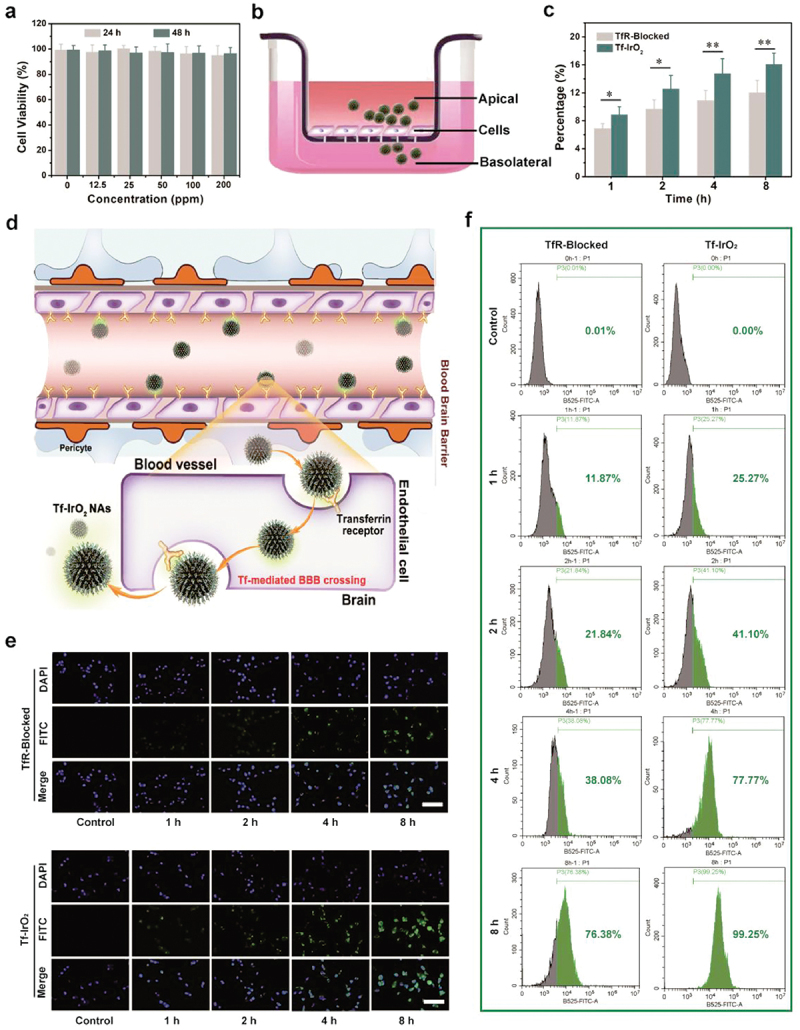
In vitro BBB crossing ability and cellular uptake of Tf-IrO_2_ NAs. (a) Cytotoxicity of HUVECs treated with various concentrations of NAs for 24 and 48 h, respectively. (b) Schematic diagram of transwell assay for cell culture of BBB model. (c) Penetrative capacity and ratio of Tf-IrO_2_ NAs across BBB with or without adding free Tf. (d) Schematic illustration of Tf-mediated BBB crossing of Tf-IrO_2_ NAs. (e) CLSM images of HUVECs after incubation with Tf-IrO_2_ NAs with or without the addition of free Tf for 1, 2, 4 and 8 h, respectively. Scale bar = 50 µm (f) flow cytometric analysis on cellular FITC levels within HUVECs after incubation with Tf-IrO_2_ with or without adding free Tf [86]. copyright 2023, Elsevier.

As for Tf, Recently, Du et al. engineered iridium oxide nano-agglomerates with Tf shell using biomineralization approach for treating cerebral ischemia-reperfusion injury. In transwell assays simulating the BBB, these Tf-coated nano-agglomerates efficiently crossed the barrier, leveraging Tf receptors (TfR) on endothelial cells. In vivo fluorescence imaging further confirmed their superior BBB penetration and brain-targeted delivery compared to uncoated nano-agglomerates (Figure 3) [86]. Prakash et al. developed holo-Tf conjugated nanomicelles to target TfR1, which is overexpressed on the endothelial cells of the BBB, particularly in ischemic regions. In vivo BBB penetration was evaluated using DiR-loaded nanomicelles injected into the common carotid artery of a middle cerebral artery occlusion (MCAO) rat model. IVIS imaging showed significantly higher fluorescence in the ischemic brain regions of treated rats compared to controls, confirming successful BBB crossing and targeted delivery to the ischemic area [87]. Similarly, Ramalho et al. designed Tf-conjugated nanoparticles for co-delivery of temozolomide and bortezomib to treat glioblastoma. These nanoparticles utilize the TfR receptor on the BBB and glioblastoma cells for improved penetration and targeted delivery, as confirmed by competitive binding assays and fluorescence quantification [88].

Other proteins such as Apo are also reported. ApoE coated nanoparticles were proved to recognize ApoE receptors, LRP-1 and LRP-2, and exhibited efficient BBB penetration with bypassing the lysosome in endothelial cells [89]. Besides, Zhang et al. developed ApoA-I-inspired nanoscavengers to effectively cross the BBB by mimicking the natural transport pathway of ApoA-I in endothelial cells. In an in vitro transwell BBB model, these nanoscavengers successfully traversed the BBB and were endocytosed by neurons and microglia in the lower chambers. In vivo fluorescence imaging in transgenic AD mice showed rapid brain entry, peaking at 8 h post-injection. Compared to non-ApoA-I nanoscavengers, the ApoA-I-inspired version exhibited approximately twice the fluorescence signal, demonstrating superior BBB penetration [90].

In addition to these endogenous proteins, the carrier protein, crossing reacting material 197 (CRM197), can penetrate the BBB by binding to diphtheria toxin receptors, which are notably upregulated in cerebral blood vessels under conditions like gliomas, ischemic stroke, and hypoxia. Singh et al. developed CRM197-functionalized nanogels loaded with radiopharmaceuticals for brain tumor therapy. In an in vitro model co-cultured with hCMEC/D3 endothelial cells and glioblastoma cells, these nanogels demonstrated a 10% increase in intracellular fluorescence and radioactivity in glioblastoma cells, indicating enhanced transcytosis without compromising tight junction integrity. Pre-treatment with CRM197 inhibited this process, confirming the specificity of diphtheria toxin receptors-mediated transcytosis [91].

Except for enhancing brain targeting and BBB penetration, proteins modification generally offers advantages in improving in vivo biocompatibility and stability of nanocarriers. It may reduce recognition and clearance by the immune system, thereby extending their circulation time in vivo [92]. Furthermore, protein modification can increase the physical and chemical stability of nanocarriers, ensuring the efficacy and safety of the drug during transportation in vivo [93]. However, they may face challenges on production, conjugation, purification and storage stability in production, as they are large molecules with three-dimensional structures and easily process desaturation, aggregation and degradation [94]. These instability issues can compromise the efficacy, safety, and shelf-life of protein-modified nanocarriers.

#### 3.1.2. Peptides

To avoid the shortages of protein modification, engineered peptides such as Angiopep-2 (AP2), Tf-peptides and ApoE peptides, were explored for RMT in the BBB penetration. As the advantages, these peptides are immunogenic, easy to synthesize, and amenable to different chemical manipulation, providing versatility in design of brain targeting nanocarriers. In several reports, Tf-peptides or ApoE peptides only with approximately 10 animo acids can efficiently recognize their corresponding receptors, TfR and LRP-1, similarly as Tf and ApoE proteins [95–98].

AP-2 is a peptide that consists of 19 amino acids with the sequence TFFYGGSRGKRNNFKTEEY, engineered to facilitate the transport of therapeutic agents across the BBB via LRP1 that abundantly expressed on BBB endothelial cells [99]. Jiang et al. proved that AP-2 modified nanocarriers exhibited successful BBB crossing with effective lysosome escape and the tight injection integrity. Compared to non-modified ones, AP-2 modified nanocarriers were more effectively internalized by glioblastoma multiform cells and macrophages after crossing the BBB [100]. Galstyan et al. found that cytotoxic T-lymphocyteassociated antigen 4 (a-CTLA-4) and programmed cell death-1 (a-PD-1) loaded nano-biopolymer scaffold functionalized with AP-2 exhibited trans-BBB delivery and increased CD8^+^ T cells, NK cells and macrophages with a decrease of regulatory T cells in the brain tumor area [101]. Other results were obtained in AP-2 modified biomimetic nanocarriers developed by Zhang et al. and Liu et al. correspondingly [102–104].

**Figure 4.**
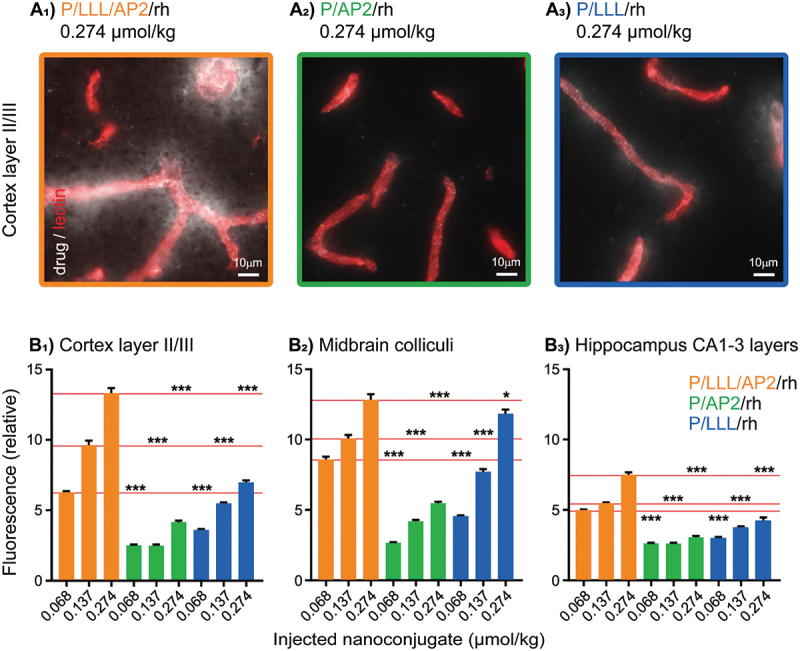
Nanoconjugate composition determines degree and locus of BBB penetration. (A_1–3_) optical imaging data showing nanoconjugate permeation of the cerebral cortex: Nanoconjugate fluorescence is gray, and the vasculature is red. Different nanoconjugates are indicated with different colors. (B) average nanoconjugate fluorescence in layers II/III of the somatosensory cortex (B_1_), the midbrain colliculi (B_2_), and the hippocampal CA1–3 cell layers (B_3_) as a function of nanoconjugate composition and concentration: P/LLL/AP2/rh is shown in red, P/AP2/rh in green, and P/LLL/rh in blue. Average nanoconjugate fluorescence measurements were obtained from 20 randomly sampled ROIs explicitly outside of the cerebral vasculature (four mice with four images each, for each measurement). Statistical tests were conducted between nanoconjugate types (*e.g*., red *vs* green) within different concentrations. The results are indicated with asterisks where * = *p* < 0.01, ** = *p* < 0.001, and *** = *p* < 0.0001; the red lines show the concentration of P/LLL/AP2/rh against which each comparison was made [105]. copyright 2019, American Chemical Society.

To further enhance the BBB penetration, Israel et al. engineered nanostructures by conjugating AP2 and trileucine (LLL), the endosome escape unit. Optical imaging demonstrated that the resulting nanoconjugate exhibited significant fluorescence in brain regions like cortical layers II/III and hippocampal CA1–3 after intravenous injection. It revealed that the BBB penetration was dose-dependent, with higher fluorescence intensity at increased concentrations, particularly near blood vessels. Removing either LLL or AP2 substantially reduced BBB penetration, underscoring their vital roles in trans-BBB delivery. Additionally, the LLL moiety enhanced the structural stability and BBB permeability of the nanoconjugate (Figure 4) [105]. This design also serves as a reference for RMT nanocarriers, particularly those modified with peptides.

In other cases, Qian et al. constructed fibroblast growth factor receptor 1(FGFR1)-ligand (FGL) peptide decorated nanoparticles to cross the BBB by utilizing FGFR1-mediated endocytosis. The nanoparticles enhanced brain accumulation of loaded drug by 4.8-fold compared to free drug. Key experiments include in vivo biodistribution studies in mice, showing peak brain concentrations at 1 h post-injection, and intracellular sorting experiments in bEnd.3 cells, demonstrating that FGL-modified nanoparticles are efficiently sorted to recycling endosomes and released at the basolateral membrane, promoting effective BBB transcytosis [106].

However, peptide-modified nanoparticles have certain limitations. Their stability in the bloodstream can be problematic since peptides are prone to degradation by proteases or easily coated by protein corona, which may diminish their effectiveness [107]. Additionally, the binding affinity of these peptides to target receptors may be compromised by endogenous ligands, resulting in competition and decreased transport efficiency [91]. Moreover, the production process and scaling up of peptide-modified nanoparticles may be challenging and expensive.

#### 3.1.3. Antibodies and engineered antibody fragments

Antibody-modified nanocarriers represent a promising strategy for drug delivery to the brain. These antibodies can be tailored to identify various targets, offering versatility in designing nanocarriers for treating different neurological conditions or brain diseases.

For instance, Aguiar et al. developed a rabbit-derived single-domain antibody (sdAb) library to target the BBB receptors using an in vivo phage display selection method. Five potential sdAbs were identified, with the most promising, RG3, being conjugated to liposomes for drug delivery. The RG3-liposomes demonstrated efficient translocation across an in vitro brain endothelial barrier (BEB) model, achieving over 0.6% ID/g in brain biodistribution studies. This confirms their ability to cross the BBB and deliver therapeutic agents effectively [108]. Other antibodies, including those targeting the TfR and other receptors, have been previously reviewed [98,109,110].

But antibody modification is considered to be disfavored in the BBB penetration of nanocarriers compared to peptides or proteins [111]. The use of antibodies modification may trigger immune responses, potentially limiting their therapeutic application, particularly with repeated dosing. Additionally, the production and purification of antibodies are both costly and technically challenging, leading to high expenses for large-scale manufacturing [112].

#### 3.1.4. Aptamers

Aptamers are short, single-stranded nucleic acids (RNA or DNA) that adopt specific three-dimensional shapes, enabling them to bind selectively and with high affinity to target receptors on endothelial cells of the BBB [113]. Aptamers offer practical benefits compared to antibodies or peptides, such as being more cost-effective, smaller in size, less immunogenic, and providing greater chemical and biological versatility. Recently, Su et al. prepared the nanoparticles camouflaged with TfR aptamers to recognize TfR and traverse the BBB via RMT. Their nanoparticles then effectively targeted microglia and neurons and promotes immune modulation in the Alzheimer’s disease brain [114].

Aptamer production faces significant challenges. Traditional Systemic Evolution of Ligands by EXponential Enrichment (SELEX) methods, while isolating high-affinity ligands, often lack physiological relevance, reducing their effectiveness in targeting native cellular receptors. Species differences in animal models further hinder translation to human use. Even advanced cell-SELEX methods, despite using in vitro human BBB models, suffer from low physiological relevance and issues like paracellular leakage, limiting the screening of aptamers with strong BBB penetration for neurological treatments.

#### 3.1.5. Other small molecules

Choline analogue 2-methacryloyloxyethyl phosphorylcholine (MPC) can be employed for decorated nanocarriers to bind with nicotinic acetylcholine receptors (nAChRs) and choline transporters, which are widely existed on the surface of BBB.

Guan et al. employed MPC-modified nanoparticles to facilitate nanoparticle delivery targeting the brain. As the result, nanoparticles with MPC exhibited significantly higher photoacoustic (PA) signal intensities in the brain compared to non-MPC nanoparticles, indicating improved BBB penetration. Quantitative analysis of PA imaging demonstrated that nanoparticles with MPC achieved the highest brain tumor accumulation, confirming effective BBB crossing and enhanced therapeutic efficacy for glioblastoma [115]. Similarly, in an orthotopic glioma model, nanoparticles with MPC loaded with Cy5 exhibited significant fluorescence in brain tumors, unlike non-MPC formulations. Quantitative analysis showed enhanced tumor accumulation and prolonged blood circulation for MPC-containing nanoparticles [116]. These reports reveal the potential application of MPC for RMT of nanocarriers for BBB penetration.

### 3.2. Carrier-mediated transport

Numerous transport systems as carriers on brain endothelial cells deliver vital nutrients and endogenous substances to the brain, distinguished by their substrate specificity. These carriers include nutrient transporters such as glucose transporters (GLUT) and amino acid transporters, ion transporters, peptide transporters, choline transporters and so on [32].

Specially, GLUT1 is overexpressed in brain capillary endothelial cells and glioblastoma, therefore, nanocarriers can be designed to target GLUT1 for improved BBB penetration and glioblastoma accumulation. Liu et al. found that their glucose-modified nanoparticles significantly enhanced transport compared to controls, as evidenced by higher fluorescence intensity of Cy7.5-labeled glucose-modified nanoparticles in brain endothelial cells and tumor spheroids, via in vitro BBB models. Transport efficiency decreased when GLUT1 activity was inhibited by phlorizin, indicating GLUT1’s role in this process. The nanoparticles also demonstrated greater internalization in tumor cells and higher antitumor effect compared to the non-glucose-modified counterpart [117]. Similarly, result was reported by Duan et al. that glucose modification could selectively target to brain capillary endothelial cells and was applicable to various nanocarriers including gold, silica, and polymeric nanoparticles [118]. For liposomes, cholesterol-undecanoate-glucose conjugate was synthesized to prepare artesunate and ligustrazine hydrochloride co-loaded liposomes for improved anticerebral malaria efficacy and brain targetability [119].

In other cases, Yang et al. employed glutathione (GSH) to modify their gold nanocages for targeting specific transporters on the blood-brain barrier, enhancing its delivery to the brain. In vitro, the modified nanocages demonstrated significant transport across a transwell BBB model, with intense fluorescence observed cells in the lower chamber, unlike non-functionalized controls. In vivo, the modified nanocages rapidly accumulated in the brain, reaching peak fluorescence at 24 h post-injection with a brain delivery efficiency of 62.48%, while non-functionalized ones showed minimal brain retention [120]. This confirms the effective BBB penetration of GSH modified nanocarriers.

Nanocarriers for carrier-mediated transport will face transport situation and endogenous substrates competition, which may limit their BBB penetration. Currently, carrier-mediated transport may more suit for brain disease with nutrient or energy metabolism impaired.

### 3.3. Cell-mediated transport

Cell-mediated transport (CMT) utilizes specific cells to aid in delivering nanocarriers via the BBB, by exploiting the inherent or modified attributes of these cells. In the following sections, CMT is introduced as three parts, including extracellular vesicles, cell membrane coating and pathogens.

#### 3.3.1. Extracellular vesicles

Extracellular vesicles (EVs) are nano-sized lipid bilayer vesicles naturally secreted by cells [121]. EVs have been identified in biological samples and cell cultures from human, non-human, and even plant and microbial origins, naturally equipped with various cell membrane proteins, facilitate membrane fusion with endothelial cells, enhancing the ability of nanocarriers to cross the BBB [122].

**Figure 5.**
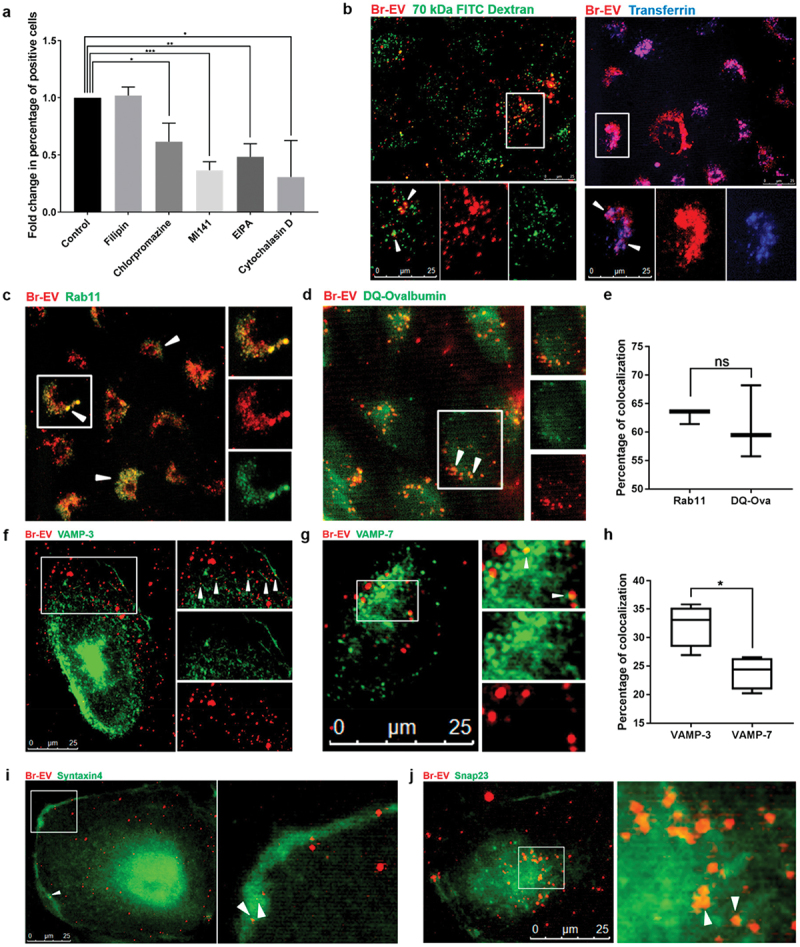
Br-EV transcytosis involves caveolin-independent endocytosis, recycling endosomes, and basolateral SNAREs. (a) Flow cytometry quantification of TdTom-br-EV uptake by brain endothelial cells in the presence of chemical inhibitors of different pathways of endocytosis (mean ± SD; 3 independent experiments). Statistical analysis was performed using unpaired two-tailed Student’s *t* test. (b) Representative fluorescence microscopy images of the colocalization of TdTom-br-EVs with 70 kDa FITC dextran (marker of macropinocytosis, left panel) and Alexa647 transferrin (marker of clathrin-dependent endocytosis, right panel) from three independent experiments. The bottom panels show magnification of the area selected by the white square. White arrows indicate colocalization. Scale bar, 25 μm. Representative fluorescence microscopy images of the colocalization of TdTom-br-EVs with (c) rab 11, (d) DQ-Ovalbumin, (f) VAMP-3, and (g) VAMP-7. The right panels show magnification of the area selected by white square. White arrows indicate colocalization. Scale bar, 25 μm. Quantification of the percentage of colocalized Br-EV-containing vesicles with rab11, DQ-Ovalbumin (e) and VAMP-3 and VAMP-7 (h) (mean ± SD; 3 independent experiments). Statistical analyses were performed using unpaired two-tailed Student’s *t* test. (i,j) Representative fluorescence microscopy images of the colocalization of TdTom-br-EVs with syntaxin 4 (i) and Snap23 (j) from three independent experiments. The right panels show magnification of the area selected by white square. White arrows indicate colocalization. Scale bar, 25 μm. In all panels, ns = not significant; *p ≤0.05; **p ≤0.01; ***p ≤0.001 [123]. copyright 2019, American Chemical Society.

For instance, Morad et al. proved that breast cancer-derived EVs could breach the BBB through transcytosis. It was confirmed using a microfluidic organ-on-a-chip model and an in vivo zebrafish model, both of which showed that breast cancer-derived EVs can traverse the BBB without disrupting its integrity. Further, breast cancer-derived EVs were identified to utilize clathrin-mediated pathways and micropinocytosis rather than caveolin-dependent endocytosis. These EVs were then sorted into recycling endosomes, specifically those marked by rab11, and being released on the basolateral side of endothelial cells through interactions with VAMP-3 and SNARE complexes (Figure 5) [123]. This pathway highlights the selective and efficient transport mechanism used by breast cancer-derived EVs to cross the BBB, which can be harnessed for developing new nanocarriers.

In other cases, Xu et al. found that mesenchymal-stem-cells derived EVs loading with Cy5.5 labeled exhibited significant brain accumulation in Alzheimer’s disease mice, with enhanced permeability observed via ex vivo imaging [124]. Macrophage derived EVs also showed improved BBB transcytosis ability than free drugs [125].

As for plant originated EVs, Niu et al. utilizes grapefruit-derived EVs combined with doxorubicin loaded heparin-based nanoparticles (DNs) to cross the blood-brain barrier (BBB). Experiments using in vitro BBB models demonstrated that EV-DNs showed significantly higher permeability efficiency than DNs or doxorubicin alone, with a 65.7% FRET efficiency, confirming their effective crossing of the BBB and targeted delivery to glioma cells [126]. Similarly, ginseng-derived EVs were proved to successfully crossed the BBB and were internalized by glioma cells, demonstrating efficient transport in a transwell BBB model [127].

EVs, especially those derived from endogenous cells, offer notable advantages due to their natural biocompatibility and reduced likelihood of eliciting strong immune responses. This makes them a safer alternative to synthetic nanocarriers and suitable for long-term therapeutic use. Despite these advantages, EVs for clinical use face significant hurdles in the process of production and purification, which involved separating, purifying, and characterizing EVs, as they are complex and costly, which hampers their widespread application. Furthermore, maintaining consistency in the vesicles’ properties and functionality throughout production is a substantial challenge.

#### 3.3.2. Cell membrane coating

Cell membrane coating is an emerging technique in the design of nanocarriers for therapeutic or diagnostic agents’ delivery across the BBB. Cell membranes contain diverse receptors and ligands that can selectively interact with matching molecules on BBB endothelial cells. This interaction triggers diapedesis [128], facilitating the passage of nanocarriers across the BBB.

For instance, Sun et al. observed that neural stem cell membrane coating facilitates nanoparticles crossing the BBB, primarily through interactions between the late antigen-4 (VLA-4) in the neural stem cell membrane and the vascular cell adhesion molecule-1 (VCAM-1) in BBB endothelial cells. After 4 h, the fluorescence intensity of membrane coated nanoparticles in the lower chamber was ~10% higher than controls with improved BBB penetration. The effectiveness was further validated as the transcytosis inhibitor VLA-4 decreased BBB penetration of membrane coated nanoparticles, emphasizing involvement neural stem cell membrane coating in the process [129]. Jia et al. also proved the enhanced BBB penetration and brain targeting of neural stem cell membrane for glioblastoma treatment [130].

As for macrophage membranes coating, macrophage membranes containing α4 and β1 integrins to cross the BBB via binding to VCAM-1 in BBB endothelial cells. Peng et al. confirmed that M1-type macrophage membrane coated nanoparticles showed the highest penetration efficiency (34.36%) among tested groups (~10%) in in vitro studies with a bEnd.3 cell monolayer [131]. Similar results were reported with enhanced BBB penetration for anti-glioblastoma [132,133].

Tumor cell membrane that expressed syndecan-1 (CD138) can bind to CD31 on endothelial cells for crossing the BBB. As the reports of He et al., in vitro assays with a transwell system showed fluorescence intensity of 4T1 cell membrane coated nanoparticles in receptor cells was 1.58-fold and 2.4-fold higher than uncoated nanoparticles at 12 and 24 h, respectively. In vivo studies in transient middle cerebral artery occlusion rat models revealed that membrane coated nanoparticles accumulated 4.79-fold more in ischemic tissue compared to the normal hemisphere and 2.88-fold more than uncoated ones, indicating effective BBB penetration and targeting of ischemic lesions [134].

Cell membrane coating improves nanocarrier biocompatibility by reducing immune detection and clearance. Since the membrane is derived from endogenous cells, it allows nanocarriers to evade immune recognition by mimicking self-cells [135,136]. Membrane coating can be optimized using cell membrane hybrids, targeting ligands, or gene transfection to enhance the expression of specific recognition proteins, thereby boosting BBB penetration and targeting efficiency [137–139]. However, scaling up production while maintaining uniformity and stability of membrane-encapsulated nanocarriers poses significant challenges. Additionally, sourcing patient-derived cell membranes require timely preparation to ensure membrane viability and functionality.

#### 3.3.3. Pathogens

In addition to EVs and membrane coating, CMT can leverage pathogens, particularly bacteria, to enhance drug delivery across the BBB. These pathogens possess inherent mechanisms enabling BBB traversal and brain entry, including bacteria or bacteria-derived outer-membrane vesicles as nanocarriers, harnessing to transport therapeutic and diagnostic agents.

**Figure 6.**
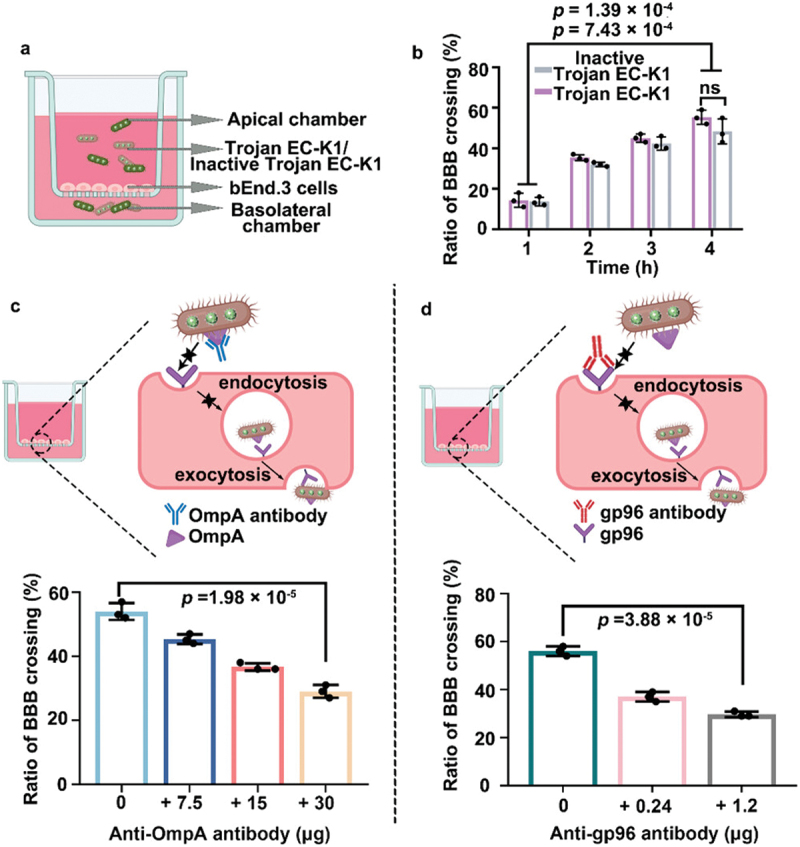
Inactive trojan EC-K1 crossing the BBB *in vitro*. (a) Scheme illustrating the construction of the *in vitro* BBB model based on bEnd.3 cells. (b) Corresponding penetration rates of trojan EC-K1 or inactive trojan EC-K1 at 1, 2, 3, and 4 h incubation. The bacterial count is ∼1.0 × 10^5^ CFU. (c) Scheme illustrating the competition experiments to confirm the uptake of inactive trojan EC-K1 by bEnd.3 cells associated OmpA and the corresponding penetration rates of inactive trojan EC-K1 in the presence of the OmpA antibody. The inactive trojan EC-K1 were first incubated with the OmpA antibody for 1.5 h and then inoculated with bEnd.3 cells. (d) Corresponding penetration rates of inactive trojan EC-K1 in the presence of the gp96 antibody. The bEnd.3 cells were first incubated with the gp96 antibody for 1.5 h and then inoculated with inactive trojan EC-K1. All imaging experiments were repeated three times with similar results. All error bars represent the standard deviation determined from three independent assays. The statistical significance is calculated via one-way analysis of variance (ANOVA) with a Tukey *post hoc* test [140]. copyright 2023, American Chemical Society.

Recently, Lu et al. utilizes inactivated *Escherichia coli* K1 (EC-K1) as nanosystems to safely cross the BBB. By maintaining the intact structure and chemotaxis of live EC-K1 while eliminating pathogenicity, the ‘dead EC-K1’ can penetrate the BBB effectively. It was revealed that the uptake of the inactive EC-K1 by brain endothelial cells was mediated through interactions between outer membrane protein A (OmpA) and its receptor gp96. In vivo experiments demonstrated that these inactivated bacteria, after intravenous injection, delivered therapeutic agents like indocyanine green (ICG) to the brain with a concentration approximately three times higher than free ICG. This capability was further validated using an in vitro BBB model, where the penetration rate of inactive EC-K1 reached 48.3% at 4 h, close to the 55.3% achieved by live EC-K1. In vivo imaging confirmed the substantial accumulation of inactivated EC-K1 in the brain, liver, and kidneys, supporting its potential as a safe and effective BBB-crossing drug delivery vehicle (Figure 6) [140].

In other cases, Pan et al. developed nanocarriers with capacity of BBB penetration by leveraging bacteria-derived outer-membrane vesicles (OMVs) that hitchhike on neutrophils. OMVs, which are recognized by neutrophil Toll-like receptors, are loaded with pioglitazone and then used to target the ischemic brain. In vitro, the nanocarriers showed a 3.2-fold increase in pioglitazone penetration with neutrophils, compared to the nanocarriers alone. In vivo studies revealed that the nanocarriers significantly accumulated in the infarcted brain, with a 3.1-fold higher fluorescence signal than free IR780, and effective neutrophil-mediated BBB traversal. Immunofluorescence and multiphoton microscopy confirm that OMVs co-localize with neutrophils in the brain, emphasizing the role of Toll-like receptor 4 (TLR4) recognition in this process [141].

Pathogens are explored less than other strategies based on CMT. Because the use of pathogenic organisms for drug delivery may introduce notable safety risks, such as potential toxicity and immune system reactions. It is essential to ensure that these organisms are made non-pathogenic while still maintaining their delivery efficiency. Additionally, pathogen-based delivery systems may encounter strict regulatory oversight due to the inherent risks, complicating both the approval and commercialization processes.

### 3.4. Extra stimuli-mediated transport

Based on the mechanisms including RMT, carrier-mediated transport, CMT, ESMT is promised to improve BBB permeability by interacting with appropriate nanocarriers. Currently, ESMT is mainly focused on ultrasound (US), magnetic field and irradiation stimulation.

#### 3.4.1. Ultrasound

Focused US (FUS) is explored as the non-invasive technique that concentrates high-intensity US waves on specific body regions such as brain. This method uses the physical properties of sound waves to converge multiple beams into a small area, creating a high-energy focal point. US stimulation enhances BBB permeability via acoustic cavitation, where microbubbles form and oscillate under ultrasound exposure. These oscillating microbubbles create mechanical forces that temporarily disrupt endothelial cell tight junctions in the BBB, increasing permeability. For instance, in an in vitro BBB model, the use of US increased the transport ratio of nano-sonosensitizer systems to 67.68%, a 1.78-fold improvement compared to those systems without US [142]. US induced BBB opening was found to be accompanied by an acute inflammatory response with the inappropriate treatment parameters such as center frequency, peak rarefractional pressure, the pulse length, the repetition frequency of the pulse and the total insonification time [143]. Therefore, US induced BBB opening will bring more clinical trials, when the questions of US treatment parameters dose will have been optimized.

Recently, Kwak et al. use FUS mediated transport to deliver biodegradable nanoparticles carrying plasmid DNA or mRNA to the brain. Results showed effective accumulation of nanoparticles in target regions and successful transgene expression in astrocytes and neurons. One experiment with nanoparticles carrying luciferase pDNA and FUS to the right striatum showed successful transgene expression after 48 h. Another with mRNA encoding mCherry demonstrated mCherry expression in astrocytes and neurons. Histopathological analysis confirmed no adverse effects, supporting the safety and efficacy of this approach for brain-targeted gene delivery [144]. Kong et al. combined FUS and engineered EVs to enhance brain delivery of CRISPRi systems. Fluorescence tracking revealed that DiR-labeled EVs showed significant brain accumulation with FUS, but increased to minimal result without FUS. In vivo, the EVs improved motor performance and reduced α-synuclein levels in Parkinson’s diseasemice. Histological and imaging analyses confirmed that EVs with FUS effectively targeted deep brain regions and restored synaptic function, validating the efficacy of the nanocarriers [145].

Furthermore, combining US with microbubble technology significantly enhances BBB permeability, improving drug delivery efficiency. This method shows great promise in experimental and preclinical studies. Wang et al. prepared a nanoprobe-loaded microbubble system for monitoring and mitigating apoptosis during FUS-mediated BBB opening. FUS induces cavitation in the microbubbles, causing temporary BBB disruption and releasing inside encapsulating the annexin V-targeted nanoprobes. These nanoprobes bind to externalized phosphatidylserine on apoptotic cells, enabling real-time detection of cell death. Experiments showed that the microbubble system successfully opened the BBB, as demonstrated by Evans blue staining and increased TUNEL-positive cells with higher acoustic pressures [146].

#### 3.4.2. Magnetic fields

Magnetic nanoparticles (MNPs) respond to external static magnetic fields (EMF), enabling researchers to direct them toward target sites, facilitating their penetration through the BBB. For instance, magnetic graphene oxide nanoparticles were observed to cross the BBB effectively under the influence of an EMF, which increased MNPs accumulation in tumor sites, as evidenced by ICP-OES analysis and MRI imaging. In vivo results showed improved BBB penetration of MNPs with magnetic field, along with tumor growth inhibition and prolonged survival of for glioma-bearing rats [147]. Similar results were reported by Chen et al. with the EMF stimulation, the bioavailability of their superparamagnetic iron oxide nanoparticles was improved over 25% compared to the group without EMF [148]. Most of nanoparticles shown that the effective passage of magnetic nanoparticles across the normal BBB in rats under an external magnetic field, with quantitative analyses showing significant accumulation in the cortex near the magnet, and progressively lower accumulation in brain tissues farther away from the magnet [149]. Hence, a single magnetic strategy is not sufficiently effective to pass the BBB. The magnetic strategy needs to be combined with others to achieve effective BBB penetration.

**Figure 7.**
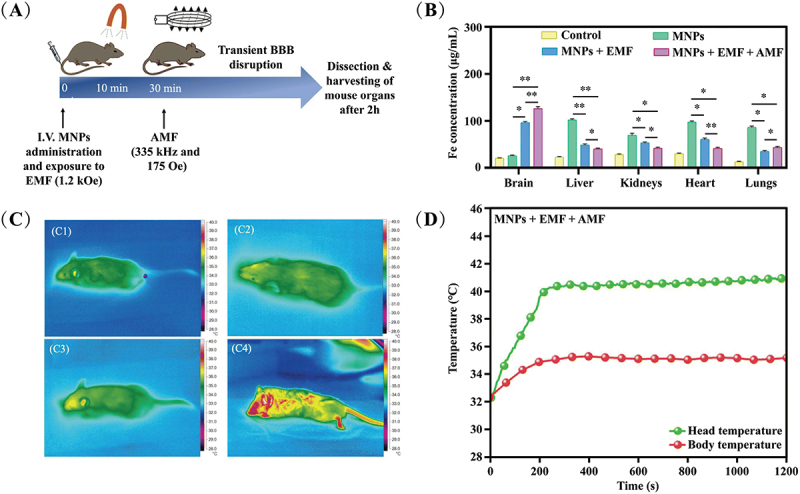
(A) Schematic diagram of the experimental design. (B) determination of Fe in murine tissues by ICP-MS. (C) thermal images of four C57BL/6 mice: (C1) PBS; (C2) MNPs; (C3) MNPs + EMF; and (C4) MNPs + EMF + AMF. (D) temperature *vs*. time curve for the group exposed to AMF. One-way ANOVA analysis was completed to determine statistical significance where *p < 0.1, **p < 0.01 were considered statistically significant [150]. copyright 2022, Royal Society of chemistry.

Besides responding to EMF, MNPs can be activated by alternating magnetic fields (AMF) to generate localized heat, raising tissue temperatures from 37°C to 42–45°C. This heat can disrupt tight junctions in the BBB, transiently increasing permeability for nanocarriers into brain tissues. Gupta et al. combine the use of an EMF to enhance cellular association with brain endothelial cells and an AMF to temporarily disrupt the tight junctions of the BBB within 3 h, allowing increased permeability for the Fe_3_O_4_ MNPs. By in vivo mouse models, dual magnetic fields targeting significantly increased MNP accumulation in the brain, as confirmed by ICP-MS iron content analysis. Biodistribution data revealed that dual magnetic targeting enhanced MNP localization in the brain while reducing non-specific distribution to other organs. Histological and toxicological assessments supported the biocompatibility of this approach, showing no significant changes in brain tissue and minimal long-term toxicity (Figure 7) [150]. Kaushik et al. presented magneto-electric nanoparticles as non-toxic, ferromagnetic, 25 ± 5 nm carriers for CRISPR-associated 9/gRNA, capable of crossing the BBB under a 0.8T EMF. Magneto-electric nanoparticles enable controlled, on-demand release via AMF, successfully reducing viral long terminal repeat levels in latent human immunodefciency virus (HIV)-infected cells [151]. Overall, this dual targeting strategy effectively improved MNPs transport across the BBB, presenting a viable method for brain therapeutic delivery.

But there are some challenges for magnetic field mediated transport. Firstly, Strength and uniformity of the applied magnetic field are crucial for nanoparticle targeting efficacy. Inadequate magnetic fields may fail to guide nanoparticles effectively, while excessive fields might cause adverse effects. Moreover, achieving a uniform magnetic field distribution, especially in deep tissues, is challenging and may result in inconsistent therapeutic outcomes. Although MNPs enhance drug delivery efficiency, their long-term biocompatibility and potential toxicity need further investigation due to possible harmful effects of degradation products. Additionally, patient variability in magnetic field strength, BBB permeability, and tumor location may lead to individualized therapeutic responses, complicating the achievement of consistent results in large-scale clinical settings.

#### 3.4.3. Irradiation

Irradiation stimulation has been explored to enhance the BBB penetration through tight junction modulation by inducing photothermal effect. As the physiologically relevant temperature rises, the permeability of the BBB increases, which is attributed to a decrease in VE-cadherin levels and the relaxation of tight junctions between neighboring endothelial cells.

Li et al. reported that picosecond laser stimulation of gold nanoparticles significantly increased BBB permeability in a graded and reversible manner. Electron microscopy revealed that lanthanum nitrate tracer filled 51% of tight junctions completely and 49% partially post-laser stimulation, compared to nearly 100% partial filling in controls. Vascular dynamics and brain parenchyma remained largely unchanged, preserving spontaneous vasomotion and structural integrity. Furthermore, this method effectively facilitated the delivery of antibodies, adeno-associated viral vectors, and liposomes into the brain, demonstrating its potential for therapeutic agent transport [152]. Du et al. proved that Nb₂C MXene-based nano-chelators activated by near-infrared (NIR)-II laser to enhance BBB permeability, with transcytosis rates rising from 1.33% to 12.49%. In vivo, Evans blue staining confirmed higher BBB penetration when combined with NIR-II laser, with an observed brain accumulation of 2.4% of Nb after 24 h [153].

## 4. Nanocarriers with synthetical mechanisms to cross BBB for brain diseases

Currently, nanocarriers have been extensively explored for brain diseases, including brain tumors, AD, PD, and brain infections (Table 2). Many of these utilize strategies previously discussed to enhance blood-brain barrier penetration, thus improving therapeutic or diagnostic outcomes. In the previous sections, different mechanisms were introduced separately, but in this section, we focus on synergistic and integrated strategies to aid the application of nanocarriers in various brain diseases. Meanwhile, nanocarriers in various brain diseases may enhance BBB permeability and induce mitochondrial damage, autophagy, neuroinflammation, and apoptosis in primary rat cortical astrocyte cells. For example, TiO_2_ NPs are likely to induce neuronal apoptosis, cognitive impairment and synaptic plasticity dysfunction in the offspring of rodents, which could also cause anxiety-like behavior, cognitive impairments, and oxidative damage in the hippocampus, particularly during the adolescent stages of neurodevelopment [163]. Hence, nanocarriers need to be particularly cautious about the potential neurotoxicity when used in the treatment of brain diseases.

**Table 2.** Nanocarriers explored for brain diseases.

Diseases		Type of Nanocarriers	Functions	Strategies to Cross the BBB	References
Brain tumors	Glioblastoma multiforme	Mature megakaryocyte membrane coated lipid nanoparticles loading with iridium photosensitizer and 8-Oxoguanine DNA glycosylase 1	1. BBB penetration and glioblastoma multiforme targeting by mature megakaryocyte membrane coating 2. Mitochondrial electron flow disruption by iridium photosensitizer and 8-Oxoguanine DNA glycosylase 1 3. ATP production inhibition and mitochondrial DNA oxidation induction 4. Immune cells recruit and intracranial antitumor immune responses activation	CMT by Mature megakaryocyte membrane coating	[154]
	Glioblastoma	AP-2 modified red blood cell membrane coated nanoparticles with polyethylenimine core and anhydride grafted poly-Llysine shell, loading with siRNA	1. Blood circulation enhancement and immune reduction by red blood cell membrane coating 2. BBB penetration by AP-2 3. Anti-glioblastoma effect by highly potent target-gene silencing by siRNA	RMT by AP-2	[155]
		Microglia membrane coating albumin nanoparticles loading with Mn-Porphyrin and anti-PD-1 antibodies	1. BBB traversal and glioblastoma microenvironment targeting by microglia membrane 2. cGAS-STING pathway activation and immune cells stimulation by Mn^2+^ 3. Tumor ablation and immunogenic cell death enhancement by NIR 4. PD-1/PD-L1 signaling axis block and immune escape inhibition by anti-PD-1	EMT by microglia membrane	[156]
	Glioma	AP-2 modified and X-ray sensitive poly(Se-Se/doxorubicin-co-acrylic acid) coated ultrasmall down-conversion nanoparticles	1. BBB penetration and glioma targeting by AP-2 2. Hydroxyl radicals’ production under X-ray irradiation 3. Doxorubicin release trigger by hydroxyl radicals 4. Fluorescence imaging under NIR-II irradiation	RMT by AP-2	[157]
		Activated mature dendritic cell membrane coated poly(lactic-co-glycolic acid) loading with rapamycin	1. BBB penetration and glioma targeting by dendritic cell membrane coating 2. CD8^+^ T cells activation and tumor immune effects promotion by dendritic cell membrane coating 3. Tumor killing by rapamycin	CMT by dendritic cell membrane coating	[128]
AD		KLVFFAED peptide modified N-doped mesoporous carbon nanospheres	1. BBB penetration and Aβ targeting by KLVFFAED peptide 2. Aβ degradation under NIR II irradiation by N-doped mesoporous carbon nanospheres 3. Reactive oxygen species clearance by superoxide dismutase and catalase activities by N-doped mesoporous carbon nanospheres	RMT by KLVFFAED peptide	[158]
		Nb_2_C MXenzyme nanosheets	1. Reactive oxygen species clearance by Nb_2_C MXenzyme nanosheets 2. Neuroinflammatory response reduction under NIR irradiation by Nb_2_C MXenzyme nanosheets	ESMT by NIR irradiation	[153]
PD		Albumin nanoparticles with Se	1. Intestinal epithelial barrier and BBB traversal by albumin 2. Damage of oxidative stress reduction 3. Mitochondrial dysfunction and apoptosis inhibition 4. Dopamine neurons protection	RMT by albumin	[159]
		Biotin modified liposomes loading with antioxidants	1. BBB penetration by biotin 2. Reactive oxygen species reduction and α-syn aggregation inhibition by antioxidants	RMT by biotin	[160]
		RVG29 modified Pt/CeO_2_ nanoparticles	1. BBB penetration and dopaminergic neurons internalization by RVG29 2. Reactive oxygen species clearance by Pt/CeO_2_ 3. Regeneration of Reactive oxygen species prevention by Pt/CeO_2_	RMT by RVG29	[161]
Brain infections	Pneumococcal meningitis	Nanoparticles composed of RVG29, Pluronic P85 unimers and bacitracin A	1. BBB penetration by RVG29 2. P-gp inhibition by Pluronic P85 unimers 3. Pneumococcal meningitis treatment by bacitracin A	RMT by RVG29	[162]
	Cerebral malaria	Glucose modified liposomes loading with artesunate and ligustrazine hydrochloride	1. BBB penetration by glucose 2. Cerebral malaria treatment by artesunate and ligustrazine hydrochloride	Carrier-mediated transport by glucose	[119]
	HIV-1	Magneto-electric nanoparticles loading with Cas9/gRNA	1. BBB traversal by EMF 2. On-demand release of Cas9/gRNA by AMF 3. Expression of long terminal repeat reduction by Cas9/gRNA	ESMT by EMF	

### 4.1. Brain tumors

By RMT and CMT, Huang et al. prepared theranostic nanovaccines for glioma based on poly(N-vinylcaprolactam) (PVCL) nanogels that incorporate cancer cell membranes functionalized with dermorphin as the ligand to cross the BBB and target glioma cells specifically. The loaded MnO_2_ and doxorubicin (DOX) could release in response to the tumor microenvironment, facilitating combined chemo- and chemodynamic therapy. In vitro assays show a 77.4% reduction in glioma cell viability, while in vivo studies using orthotopic glioma mouse models demonstrate a significant 52.6% tumor growth inhibition and a reduced recurrence rate compared to controls [164]. In other cases, DOX encapsuled-engineered EVs were functionalized with AP-2, to cross the BBB and treat the glioma, which significantly prolonged more than 2-fold survival time of glioma mice [104].

Based on RMT plus ESMT, Ge et al. developed Bi-doped Ag2S nanocrystals (NCs) with a high photoluminescence quantum yield (~13.3%) show promise for glioma imaging. These NCs, conjugated with lactoferrin, can effectively cross the BBB with the activation of NIR-II and target gliomas. They demonstrate deep tissue penetration (5–6 mm), and high biocompatibility. In vivo imaging revealed effective glioma targeting and BBB penetration, with a fluorescence peak observed at 8 h post-injection. The NCs exhibited minimal toxicity and significant stability compared to other NCs [84]. Similarly, Reichel prepared HMC-FMX nanoprobes, combining carboxymethyl dextran-coated superparamagnetic iron oxide nanoparticles (FMX) with a heptamethine carbocyanine (HMC) fluorescent ligand to recognize and bind organic anion transporter polypeptide on BBB and activated by NIR, whose fluorescence can precise tumor boundary identification. In vivo studies show high tumor fluorescence at 24 h post-injection, with minimal fluorescence in normal brain tissues, indicating effective targeting and imaging capabilities. HMC-FMX demonstrates potential for improving GBM surgery through fluorescence-guided tumor resection [165].

### 4.2. Alzheimer’s disease

Combined RMT with CMT, a novel gene therapy approach utilizes exosome-liposome hybrid nanovesicles (TSEL) to address AD. These nanovesicles, which combine β-site amyloid precursor protein cleaving enzyme-1 (BACE1) siRNA and TREM2 plasmid, efficiently cross the BBB due to exosome homing and AP-2 peptides. TSEL enhances TREM2 expression in microglia, reprogramming them from a pro-inflammatory M1 state to an anti-inflammatory M2 state, and boosts Aβ phagocytosis. Simultaneously, siBACE1 reduces Aβ plaque production. In vivo studies showed that TSEL improved cognitive function in APP/PS1 mice, reduced Aβ burden, and mitigated neuroinflammation. TSEL also exhibited low toxicity and effectively improved memory deficits, demonstrated by behavioral tests and neurobiological assessments [166]. Similarly, Huang et al. develop RVG-NV-NPs with bexarotene (Bex) and AgAuSe quantum dots encapsulated in neural stem cell (NSC) membranes overexpressing Lamp2b-rabies virus glycoprotein (RVG). The RVG targets AChR on cerebrovascular endothelial and nerve cells, while the NSC membranes provide brain-homing properties, which facilitates BBB penetration. The RVG-NV-NPs showed a 40% reduction in Aβ levels and significant neuronal protection in AD models compared to oral Bex, highlighting improved therapeutic efficacy due to enhanced BBB permeability and targeted delivery [167].

For the combination between RMT and ESMT, Han et al. introduced a novel approach for photoacoustic imaging of brain copper levels in AD using ZnSe nanoplatelets. These ultrathin nanoplatelets undergo in situ cation exchange with endogenous copper ions to form CuSe, enhancing NIR absorption and photoacoustic signals. Modified with an AP-2 peptide for BBB targeting, the ZnSe nanoplatelets more efficiently cross the BBB and facilitate high-contrast imaging of brain copper levels. In vivo tests demonstrated significant PA signal enhancement in AD mice compared to normal mice, with imaging contrast increasing up to 3.6 times. Additionally, these nanoplatelets exhibited strong ROS inhibition, reducing neuronal apoptosis and oxidative stress. The data underscores their potential for studying brain copper dynamics and AD pathology [168].

### 4.3. Parkinson’s disease

For instance, Single-atom catalysts (SACs) Pt/CeO₂ encapsulated in HL-60 cell membranes and modified with RVG29 demonstrated superior BBB penetration, which effectively degraded reactive oxygen species (ROS) and prevented their regeneration by disrupting the α-glycerophosphate and malate-aspartate shuttle pathways, leading to mitophagy and removal of dysfunctional mitochondria. Behavioral tests showed that RVG29@AHM@Pt/CeO₂ significantly improved motor performance that mice exhibited increased fall latency (rotarod test), reduced climbing time (pole test), and enhanced movement distance (open field test) compared to the PD group and other treatments. The number of tyrosine hydroxylase (TH)-positive cells in the striatum increased and terminal deoxynucleotidyl transferase-mediated dUTP nick end labeling (TUNEL) staining revealed fewer apoptotic cells in the RVG29@AHM@Pt/CeO₂-treated mice, indicating improved neuroprotection. Additionally, RVG29@AHM@Pt/CeO₂ treatment reduced pro-inflammatory cytokines IL-6, TNF-α, and IL-1β to levels comparable to the control group [161]. Other nanocarriers employing single strategies to cross the BBB have also been used for the treatment of PD [159,160,169], but they are not the focus of this section.

### 4.4. Brain infections

Chen et al. developed a virus-mimicking NIR-II nanoprobe, composed of Fe^2+^-coordinated vesicles encapsulating PbS quantum dots, with surfaces modified by GM1 and Japanese encephalitis virus (JEV) E protein, enhancing BBB permeability via RMT and ESMT to target neuronal cells. The nanoprobe’s fluorescence, regulated by Fe^2+^/Fe^3+^ redox cycling, enables real-time, reversible detection of ROS/RNS during viral encephalitis. In vivo, lipopolysaccharides-treated mice showed significant fluorescence enhancement in the brain, with an S/N ratio exceeding 8 within 10 min post-injection. Additionally, during JEV-induced encephalitis, fluorescence peaked on the third day, correlating with increased ROS/RNS levels and viral replication [170]. These findings highlight the nanoprobe’s potential for clinical imaging applications.

For CMT plus ESMT, Shi et al. developed non-inflammatory magnetic outer-membrane vesicles (OMVs) derived from a CRISPR/Cas9-engineered Escherichia coli strain, where lipid A acyltransferase synthesis was inhibited. These OMVs were loaded with ceftriaxone (CRO) and meso-tetra-(4-carboxyphenyl)porphine and magnetically driven across the blood-brain barrier (BBB). In a mouse model of ceftriaxone-tolerant E. coli meningitis, the OMVs achieved a 20-fold greater reduction in bacterial CFUs in the brain compared to free CRO, following magnetic targeting and ultrasound application. This method significantly improved survival rates, reduced brain damage, and minimized the risk of infection recurrence [171].

## 5. Conclusion and outlooks

As the protective interface between blood and central nervous system, the BBB prevents approximately 98% of small molecule drugs and nearly all large molecules from entering the brain tissue. It is composed of a single layer of endothelial cells with tight injection, which is enveloped by pericytes and wrapped in the basement membrane and astrocytic end-feet. Current methods to bypass the BBB, such as invasive procedures, are fraught with risks like infection and brain damage, and are not suitable for long-term treatment.

Nanocarriers provide opportunities to cross the BBB for treatment and diagnosis of brain diseases, including tumors, AD, PD and infections, with several mechanisms have been reported. In detail, ligand modified nanocarriers could recognize and bind the overpressed receptors on the endothelial cells for transcytosis as RMT, such as Tf and TfR or AP-2 and LRP1. Glucose or GSH modified nanocarriers can cross the endothelial cells layer via the assistance of transporters, as carrier-mediated transport. Moreover, biomimetic nanocarriers such as EVs or cell membrane coated nanoparticles penetrate the BBB by exploiting the inherent or modified attributes of these cells as CMT. Additionally, extra-stimulation helps nanocarriers to cross the barrier via interrupting the tight injection or controlling the movement of nanoparticles. In preclinical researches, RMT and CMT or ESMT are often combined to improve the BBB traversal of these nanocarriers in brain diseases.

For the in vivo behavior of nanocarriers, the influences of protein corona may be ignored. After nanocarriers enter the blood, mono or multi-layers of proteins will adsorb on their surface, as protein corona [172]. Therefore, the modified nanocarriers for RMT or carrier-mediated transport may be affected to bind to receptors or transporters, thus reducing BBB penetration, as their ligands may be wrapped by these proteins. But protein corona also provides opportunities for unfunctionalized nanocarriers to cross the BBB via RMT. Recently, mesoporous silica nanoparticles and serum EVs were reported to capture ApoE and Tf correspondingly in circulation, which further promoted the efficiency to cross the BBB [122,173].

For the improvement BBB penetration capacity of nanocarriers, traditional Chinese medicines are taken into consideration. For instance, aromatic traditional Chinese medicines, including borneol, musk and mint, have been reported to enhance the BBB permeability, which may be useful for the treatment and diagnosis of brain tumors, AD and PD [174]. But most of traditional Chinese medicines exhibited with unfavorable physicochemical properties such as poor water‐solubility and stability, fast systemic clearance with short half‐life, and large molecular size which hampers their ability to reach the brain [175]. Therefore, they can be loaded in nanoparticles to avoid these properties, promoting the BBB traversal of nanocarriers.

Currently, most research on nanocarriers for crossing the BBB remains in the exploratory phase, with few reaching clinical trials. To accelerate the translation of these technologies from bench to bedside, the design of nanocarriers must prioritize simplicity, scalability, high efficiency, and low toxicity. These considerations are crucial for advancing the clinical application of nanocarriers in the treatment and diagnosis of brain diseases.

## Data Availability

No datasets were generated or analyzed during the current study.

## References

[1] Terstappen GC, Meyer AH, Bell RD, et al. Strategies for delivering therapeutics across the blood-brain barrier. Nat Rev Drug Discov. 2021;20(5):362–26. doi: 10.1038/s41573-021-00139-y 33649582

[2] Zhao Z, Nelson AR, Betsholtz C, et al. Establishment and dysfunction of the blood-brain barrier. Cell. 2015;163(5):1064–1078. doi: 10.1016/j.cell.2015.10.067 26590417 PMC4655822

[3] Upton DH, Ung C, George SM, et al. Challenges and opportunities to penetrate the blood-brain barrier for brain cancer therapy. Theranostics. 2022;12(10):4734–4752. doi: 10.7150/thno.69682 35832071 PMC9254248

[4] Furtado D, Bjornmalm M, Ayton S, et al. Overcoming the blood-brain barrier: the role of nanomaterials in treating neurological diseases. Adv Mater. 2018;30(46):e1801362. doi: 10.1002/adma.201801362 30066406

[5] Wu D, Chen Q, Chen X, et al. The blood-brain barrier: structure, regulation, and drug delivery. Signal Transduct Target Ther. 2023;8(1):217. doi: 10.1038/s41392-023-01481-w 37231000 PMC10212980

[6] Pandit R, Chen L, Gotz J. The blood-brain barrier: physiology and strategies for drug delivery. Adv Drug Deliv Rev. 2020;165–166:1–14. doi: 10.1016/j.addr.2019.11.009 31790711

[7] Song X, Qian H, Yu Y. Nanoparticles mediated the diagnosis and therapy of glioblastoma: bypass or cross the blood-brain barrier. Small. 2023;19(45):e2302613. doi: 10.1002/smll.202302613 37415556

[8] Lochhead JJ, Thorne RG. Intranasal delivery of biologics to the central nervous system. Adv Drug Deliv Rev. 2012;64(7):614–628. doi: 10.1016/j.addr.2011.11.002 22119441

[9] Lim SH, Yee GT, Khang D. Nanoparticle-based combinational strategies for overcoming the blood-brain barrier and blood-tumor barrier. Int J Nanomed. 2024;19:2529–2552. doi: 10.2147/IJN.S450853 PMC10949308 38505170

[10] Dadfar SM, Roemhild K, Drude NI, et al. Iron oxide nanoparticles: diagnostic, therapeutic and theranostic applications. Adv Drug Deliv Rev. 2019;138:302–325. doi: 10.1016/j.addr.2019.01.005 30639256 PMC7115878

[11] Mitchell MJ, Billingsley MM, Haley RM, et al. Engineering precision nanoparticles for drug delivery. Nat Rev Drug Discov. 2021;20(2):101–124. doi: 10.1038/s41573-020-0090-8 33277608 PMC7717100

[12] Zha S, Liu H, Li H, et al. Functionalized nanomaterials capable of crossing the blood-brain barrier. ACS Nano. 2024;18(3):1820–1845. doi: 10.1021/acsnano.3c10674 38193927 PMC10811692

[13] Tang W, Fan W, Lau J, et al. Emerging blood-brain-barrier-crossing nanotechnology for brain cancer theranostics. Chem Soc Rev. 2019;48(11):2967–3014. doi: 10.1039/C8CS00805A 31089607

[14] Pallares RM, Mottaghy FM, Schulz V, et al. Nanoparticle diagnostics and theranostics in the clinic. J Nucl Med. 2022;63(12):1802–1808. doi: 10.2967/jnumed.122.263895 36302654 PMC9730918

[15] Application of nanoparticles for cyclic hyperthermia in adjuvant therapy of glioblastoma multiforme (ANCHIALE). Available from: https://clinicaltrials.gov/study/NCT06271421?cond=glioblastoma&intr=nanoparticle&rank=2&tab=table

[16] Nu-0129 in treating patients with recurrent glioblastoma or gliosarcoma undergoing surgery. Available from: https://clinicaltrials.gov/study/NCT03020017?cond=glioblastoma&intr=nanoparticle&rank=3

[17] A study to evaluate the safety, tolerability and immunogenicity of EGFR(V)-EDV-Dox in subjects with recurrent glioblastoma multiforme (GBM) (CerebralEDV). Available from: https://clinicaltrials.gov/study/NCT02766699?cond=glioblastoma&intr=nanoparticle&rank=4&tab=results

[18] Mtx110 by convection-enhanced delivery in treating participants with newly-diagnosed diffuse intrinsic pontine glioma (PNOC015). Available from: https://clinicaltrials.gov/study/NCT03566199?cond=brain%20tumor&intr=nanoparticle&rank=6&tab=results

[19] Radiosensitization of multiple brain metastases using AGuIX gadolinium based nanoparticles (NANO-RAD). Available from: https://clinicaltrials.gov/study/NCT02820454?cond=brain%20tumor&intr=nanoparticle&rank=4&tab=results

[20] Verry C, Sancey L, Dufort S, et al. Treatment of multiple brain metastases using gadolinium nanoparticles and radiotherapy: nANO-RAD, a phase I study protocol. BMJ Open. 2019;9(2):e023591. doi: 10.1136/bmjopen-2018-023591 PMC6377538 30755445

[21] Aguix nanoparticles with radiotherapy plus concomitant temozolomide in the treatment of newly diagnosed glioblastoma (NANO-GBM). Available from: https://clinicaltrials.gov/study/NCT04881032?cond=glioblastoma&intr=nanoparticle&rank=1

[22] Thivat E, Casile M, Moreau J, et al. Phase I/II study testing the combination of AGuIX nanoparticles with radiochemotherapy and concomitant temozolomide in patients with newly diagnosed glioblastoma (NANO-GBM trial protocol). BMC Cancer. 2023;23(1):344. doi: 10.1186/s12885-023-10829-y 37060055 PMC10105392

[23] Radiotherapy of multiple brain metastases using AGuIX® (NANORAD2). Available from: https://clinicaltrials.gov/study/NCT03818386?cond=brain%20tumor&intr=nanoparticle&rank=1&tab=results

[24] Evaluating AGuIX® nanoparticles in combination with stereotactic radiation for brain metastases (NANOSTEREO). Available from: https://clinicaltrials.gov/study/NCT04094077?cond=brain%20tumor&intr=nanoparticle&rank=3&tab=results

[25] Stereotactic brain-directed radiation with or without Aguix gadolinium-based nanoparticles in brain metastases. Available from: https://clinicaltrials.gov/study/NCT04899908?cond=brain%20tumor&intr=nanoparticle&rank=2

[26] Study of APH-1105 in patients with mild to moderate Alzheimer’s disease. Available from: https://clinicaltrials.gov/study/NCT03806478?cond=Alzheimer%27s%20Disease&intr=nanoparticle&rank=1&tab=results

[27] 31P-MRS imaging to assess the effects of CNM-Au8 on impaired neuronal redox state in Parkinson’s disease (REPAIR-PD). Available from: https://clinicaltrials.gov/study/NCT03815916?cond=Parkinson%27s%20disease&intr=nanoparticle&rank=1&tab=results

[28] Australasian nanoparticle-mediated magnetically enhanced diffusion for ischemic stroke (AusNanoMED). Available from: https://clinicaltrials.gov/study/NCT06495671?cond=Stroke&intr=nanoparticle&rank=1&tab=results

[29] Pulse endovascular reperFUSION for acute ischemic stroke (PERFUSION AIS). Available from: https://clinicaltrials.gov/study/NCT06052969?cond=Stroke&intr=nanoparticle&rank=3&tab=table

[30] Langen UH, Ayloo S, Gu C. Development and cell biology of the blood-brain barrier. Annu Rev Cell Dev Biol. 2019;35(1):591–613. doi: 10.1146/annurev-cellbio-100617-062608 31299172 PMC8934576

[31] Wettschureck N, Strilic B, Offermanns S. Passing the vascular barrier: endothelial signaling processes controlling extravasation. Physiol Rev. 2019;99(3):1467–1525. doi: 10.1152/physrev.00037.2018 31140373

[32] Kadry H, Noorani B, Cucullo L. A blood-brain barrier overview on structure, function, impairment, and biomarkers of integrity. Fluids Barriers CNS. 2020;17(1):69. doi: 10.1186/s12987-020-00230-3 33208141 PMC7672931

[33] Boye K, Geraldo LH, Furtado J, et al. Endothelial Unc5B controls blood-brain barrier integrity. Nat Commun. 2022;13(1):1169. doi: 10.1038/s41467-022-28785-9 35246514 PMC8897508

[34] Greene C, Hanley N, Reschke CR, et al. Microvascular stabilization via blood-brain barrier regulation prevents seizure activity. Nat Commun. 2022;13(1):2003. doi: 10.1038/s41467-022-29657-y 35422069 PMC9010415

[35] Brandon KD, Frank WE, Stroka KM. Junctions at the crossroads: the impact of mechanical cues on endothelial cell-cell junction conformations and vascular permeability. Am J Physiol Cell Physiol. 2024;327(4):C1073–C1086. doi: 10.1152/ajpcell.00605.2023 39129490 PMC11481987

[36] Zhang TT, Li W, Meng G, et al. Strategies for transporting nanoparticles across the blood-brain barrier. Biomater Sci. 2016;4(2):219–229. doi: 10.1039/C5BM00383K 26646694

[37] Balda MS, Matter K. Tight junctions. Curr Biol. 2023;33(21):R1135–R1140. doi: 10.1016/j.cub.2023.09.027 37935122

[38] Abumrad NA, Cabodevilla AG, Samovski D, et al. Endothelial cell receptors in tissue lipid uptake and metabolism. Circ Res. 2021;128(3):433–450. doi: 10.1161/CIRCRESAHA.120.318003 33539224 PMC7959116

[39] Ayloo S, Gu C. Transcytosis at the blood-brain barrier. Curr Opin Neurobiol. 2019;57:32–38. doi: 10.1016/j.conb.2018.12.014 30708291 PMC6629499

[40] Veys K, Fan Z, Ghobrial M, et al. Role of the GLUT1 glucose transporter in postnatal CNS angiogenesis and blood-brain barrier integrity. Circ Res. 2020;127(4):466–482. doi: 10.1161/CIRCRESAHA.119.316463 32404031 PMC7386868

[41] Lee HW, Xu Y, Zhu X, et al. Endothelium-derived lactate is required for pericyte function and blood-brain barrier maintenance. Embo J. 2022;41(9):e109890. doi: 10.15252/embj.2021109890 35243676 PMC9058541

[42] Banks WA. The blood-brain barrier as an endocrine tissue. Nat Rev Endocrinol. 2019;15(8):444–455. doi: 10.1038/s41574-019-0213-7 31127254

[43] Zierfuss B, Larochelle C, Prat A. Blood-brain barrier dysfunction in multiple sclerosis: causes, consequences, and potential effects of therapies. Lancet Neurol. 2024;23(1):95–109. doi: 10.1016/S1474-4422(23)00377-0 38101906

[44] Denes A, Hansen CE, Oezorhan U, et al. Endothelial cells and macrophages as allies in the healthy and diseased brain. Acta NeuroPathol. 2024;147(1):38. doi: 10.1007/s00401-024-02695-0 38347307 PMC10861611

[45] Galea I. The blood-brain barrier in systemic infection and inflammation. Cell Mol Immunol. 2021;18(11):2489–2501. doi: 10.1038/s41423-021-00757-x 34594000 PMC8481764

[46] Armulik A, Genove G, Mae M, et al. Pericytes regulate the blood-brain barrier. Nature. 2010;468(7323):557–561. doi: 10.1038/nature09522 20944627

[47] Mae MA, He L, Nordling S, et al. Single-cell analysis of blood-brain barrier response to pericyte loss. Circ Res. 2021;128(4):e46–e62. doi: 10.1161/CIRCRESAHA.120.317473 33375813 PMC10858745

[48] Ayloo S, Lazo CG, Sun S, et al. Pericyte-to-endothelial cell signaling via vitronectin-integrin regulates blood-CNS barrier. Neuron. 2022;110(10):1641–1655. doi: 10.1016/j.neuron.2022.02.017 35294899 PMC9119930

[49] Singh A, Veeriah V, Xi P, et al. Angiocrine signals regulate quiescence and therapy resistance in bone metastasis. JCI Insight. 2019;4(13). doi: 10.1172/jci.insight.125679 PMC6629249 31292293

[50] Dave JM, Chakraborty R, Agyemang A, et al. Loss of TGFbeta-mediated repression of Angiopoietin-2 in pericytes underlies germinal matrix hemorrhage pathogenesis. Stroke. 2024;55(9):2340–2352. doi: 10.1161/STROKEAHA.123.045248 39129597 PMC11347087

[51] Knox EG, Aburto MR, Clarke G, et al. The blood-brain barrier in aging and neurodegeneration. Mol Psychiatry. 2022;27(6):2659–2673. doi: 10.1038/s41380-022-01511-z 35361905 PMC9156404

[52] Yao Y. Basement membrane and stroke. J Cereb Blood Flow Metab. 2019;39(1):3–19. doi: 10.1177/0271678X18801467 30226080 PMC6311666

[53] Schevenels G, Cabochette P, America M, et al. A brain-specific angiogenic mechanism enabled by tip cell specialization. Nature. 2024;628(8009):863–871. doi: 10.1038/s41586-024-07283-6 38570687 PMC11041701

[54] Halder SK, Sapkota A, Milner R. The impact of genetic manipulation of laminin and integrins at the blood-brain barrier. Fluids Barriers CNS. 2022;19(1):50. doi: 10.1186/s12987-022-00346-8 35690759 PMC9188059

[55] Trout AL, Rutkai I, Biose IJ, et al. Review of alterations in perlecan-associated vascular risk factors in dementia. Int J Mol Sci. 2020;21(2):679. doi: 10.3390/ijms21020679 31968632 PMC7013765

[56] Vymola P, Garcia-Borja E, Cervenka J, et al. Fibrillar extracellular matrix produced by pericyte-like cells facilitates glioma cell dissemination. Brain Pathol. 2024;34(6):e13265. doi: 10.1111/bpa.13265 38705944 PMC11483521

[57] Keeley DP, Sherwood DR. Tissue linkage through adjoining basement membranes: the long and the short term of it. Matrix Biol. 2019;75–76:58–71. doi: 10.1016/j.matbio.2018.05.009 PMC6252152 29803937

[58] Xu L, Nirwane A, Yao Y. Basement membrane and blood-brain barrier. Stroke Vasc Neurol. 2019;4(2):78–82. doi: 10.1136/svn-2018-000198 31338215 PMC6613871

[59] Manu DR, Slevin M, Barcutean L, et al. Astrocyte involvement in blood-brain barrier function: a critical update highlighting novel, complex, neurovascular interactions. Int J Mol Sci. 2023;24(24):17146. doi: 10.3390/ijms242417146 38138976 PMC10743219

[60] Diaz-Castro B, Robel S, Mishra A. Astrocyte endfeet in brain function and pathology: open questions. Annu Rev Neurosci. 2023;46(1):101–121. doi: 10.1146/annurev-neuro-091922-031205 36854317

[61] Abbott NJ, Ronnback L, Hansson E. Astrocyte-endothelial interactions at the blood-brain barrier. Nat Rev Neurosci. 2006;7(1):41–53. doi: 10.1038/nrn1824 16371949

[62] Song B, Wang X, Qin L, et al. Brain gliomas: diagnostic and therapeutic issues and the prospects of drug-targeted nano-delivery technology. Pharmacol Res. 2024;206:107308. doi: 10.1016/j.phrs.2024.107308 39019336

[63] Genovesi LA, Puttick S, Millar A, et al. Patient-derived orthotopic xenograft models of medulloblastoma lack a functional blood-brain barrier. Neuro Oncol. 2021;23(5):732–742. doi: 10.1093/neuonc/noaa266 33258962 PMC8099473

[64] van Tellingen O, Yetkin-Arik B, de Gooijer MC, et al. Overcoming the blood-brain tumor barrier for effective glioblastoma treatment. Drug Resist Updat. 2015;19:1–12. doi: 10.1016/j.drup.2015.02.002 25791797

[65] Scheltens P, De Strooper B, Kivipelto M, et al. Alzheimer’s disease. Lancet. 2021;397(10284):1577–1590. doi: 10.1016/S0140-6736(20)32205-4 33667416 PMC8354300

[66] Monteiro AR, Barbosa DJ, Remiao F, et al. Alzheimer’s disease: insights and new prospects in disease pathophysiology, biomarkers and disease-modifying drugs. Biochem Pharmacol. 2023;211:115522. doi: 10.1016/j.bcp.2023.115522 36996971

[67] Jucker M, Walker LC. Alzheimer’s disease: from immunotherapy to immunoprevention. Cell. 2023;186(20):4260–4270. doi: 10.1016/j.cell.2023.08.021 37729908 PMC10578497

[68] Tolosa E, Garrido A, Scholz SW, et al. Challenges in the diagnosis of Parkinson’s disease. Lancet Neurol. 2021;20(5):385–397. doi: 10.1016/S1474-4422(21)00030-2 33894193 PMC8185633

[69] Weintraub D, Aarsland D, Chaudhuri KR. The neuropsychiatry of Parkinson’s disease: advances and challenges. Lancet Neurol. 2022;21(1):89–102. doi: 10.1016/S1474-4422(21)00330-6 34942142 PMC8800169

[70] Cheng G, Liu Y, Ma R, et al. Anti-parkinsonian therapy: strategies for crossing the blood-brain barrier and nano-biological effects of nanomaterials. Nanomicro Lett. 2022;14(1):105. doi: 10.1007/s40820-022-00847-z 35426525 PMC9012800

[71] Bloem BR, Okun MS, Klein C. Parkinson’s disease. Lancet. 2021;397(10291):2284–2303. doi: 10.1016/S0140-6736(21)00218-X 33848468

[72] Nau R, Blei C, Eiffert H. Intrathecal antibacterial and antifungal therapies. Clin Microbiol Rev. 2020;33(3). doi: 10.1128/CMR.00190-19 PMC7194852 32349999

[73] Lv W, Liu Y, Li S, et al. Advances of nano drug delivery system for the theranostics of ischemic stroke. J Nanobiotechnol. 2022;20(1):248. doi: 10.1186/s12951-022-01450-5 PMC9153106 35641956

[74] Parvez S, Kaushik M, Ali M, et al. Dodging blood brain barrier with “nano” warriors: novel strategy against ischemic stroke. Theranostics. 2022;12(2):689–719. doi: 10.7150/thno.64806 34976208 PMC8692911

[75] Li X, Han Z, Wang T, et al. Cerium oxide nanoparticles with antioxidative neurorestoration for ischemic stroke. Biomaterials. 2022;291:121904. doi: 10.1016/j.biomaterials.2022.121904 36403323

[76] Zhu TT, Wang H, Gu HW, et al. Melanin-like polydopamine nanoparticles mediating anti-inflammatory and rescuing synaptic loss for inflammatory depression therapy. J Nanobiotechnol. 2023;21(1):52. doi: 10.1186/s12951-023-01807-4 PMC9913011 36765377

[77] Manzanares D, Cena V. Endocytosis: the nanoparticle and submicron nanocompounds gateway into the cell. Pharmaceutics. 2020;12(4):371. doi: 10.3390/pharmaceutics12040371 32316537 PMC7238190

[78] Yang C, He B, Dai W, et al. The role of caveolin-1 in the biofate and efficacy of anti-tumor drugs and their nano-drug delivery systems. Acta Pharm Sin B. 2021;11(4):961–977. doi: 10.1016/j.apsb.2020.11.020 33996409 PMC8105775

[79] Donahue ND, Acar H, Wilhelm S. Concepts of nanoparticle cellular uptake, intracellular trafficking, and kinetics in nanomedicine. Adv Drug Deliv Rev. 2019;143:68–96. doi: 10.1016/j.addr.2019.04.008 31022434

[80] Liu HJ, Xu P. Strategies to overcome/penetrate the BBB for systemic nanoparticle delivery to the brain/brain tumor. Adv Drug Deliv Rev. 2022;191:114619. doi: 10.1016/j.addr.2022.114619 36372301 PMC9724744

[81] Janjua TI, Ahmed-Cox A, Meka AK, et al. Facile synthesis of lactoferrin conjugated ultra small large pore silica nanoparticles for the treatment of glioblastoma. Nanoscale. 2021;13(40):16909–16922. doi: 10.1039/D1NR03553C 34533167

[82] Wang R, Song W, Zhu J, et al. Biomimetic nano-chelate diethyldithiocarbamate Cu/Fe for enhanced metalloimmunity and ferroptosis activation in glioma therapy. J Control Release. 2024;368:84–96. doi: 10.1016/j.jconrel.2024.02.004 38331004

[83] Li L, Lu Y, Xu X, et al. Catalytic-enhanced lactoferrin-functionalized Au-Bi(2) Se(3) nanodots for Parkinson’s disease therapy via reactive oxygen attenuation and mitochondrial protection. Adv Healthc Mater. 2021;10(13):e2100316. doi: 10.1002/adhm.202100316 34050627

[84] Ge W, Chen G, Huang X, et al. Heteroions radii matching produced intensely luminescent bismuth-Ag(2)S nanocrystals for through-skull NIR-II imaging of orthotopic glioma. Nano Lett. 2024;24(15):4562–4570. doi: 10.1021/acs.nanolett.4c00604 38591327

[85] Guo W, Ji M, Li Y, et al. Iron ions-sequestrable and antioxidative carbon dot-based nano-formulation with nitric oxide release for Parkinson’s disease treatment. Biomaterials. 2024;309:122622. doi: 10.1016/j.biomaterials.2024.122622 38797119

[86] Du W, Wang J, Zhou L, et al. Transferrin-targeted iridium nanoagglomerates with multi-enzyme activities for cerebral ischemia-reperfusion injury therapy. Acta biomater. 2023;166:524–535. doi: 10.1016/j.actbio.2023.04.025 37088161

[87] Prakash R, Vyawahare A, Sakla R, et al. Nlrp3 inflammasome-targeting nanomicelles for preventing ischemia-reperfusion-induced inflammatory injury. ACS Nano. 2023;17(9):8680–8693. doi: 10.1021/acsnano.3c01760 37102996

[88] Ramalho MJ, Torres ID, Loureiro JA, et al. Transferrin-conjugated PLGA nanoparticles for co-delivery of temozolomide and bortezomib to glioblastoma cells. ACS Appl Nano Mater. 2023;6(15):14191–14203. doi: 10.1021/acsanm.3c02122 37588263 PMC10426337

[89] Wunsch A, Mulac D, Langer K. Lipoprotein imitating nanoparticles: lecithin coating binds ApoE and mediates non-lysosomal uptake leading to transcytosis over the blood-brain barrier. Int J Pharm. 2020;589:119821. doi: 10.1016/j.ijpharm.2020.119821 32861770

[90] Zhang H, Jiang W, Zhao Y, et al. Lipoprotein-inspired nanoscavenger for the three-pronged modulation of microglia-derived neuroinflammation in Alzheimer’s disease therapy. Nano Lett. 2022;22(6):2450–2460. doi: 10.1021/acs.nanolett.2c00191 35271279

[91] Singh S, Drude N, Blank L, et al. Protease responsive nanogels for transcytosis across the blood-brain barrier and intracellular delivery of radiopharmaceuticals to brain tumor cells. Adv Healthc Mater. 2021;10(20):e2100812. doi: 10.1002/adhm.202100812 34490744 PMC11468667

[92] Bhargava A, Dev A, Mohanbhai SJ, et al. Pre-coating of protein modulate patterns of corona formation, physiological stability and cytotoxicity of silver nanoparticles. Sci Total Environ. 2021;772:144797. doi: 10.1016/j.scitotenv.2020.144797 33578167

[93] Li C, Zhou L, Yin X. Pathophysiological aspects of transferrin-a potential nano-based drug delivery signaling molecule in therapeutic target for varied diseases. Front Pharmacol. 2024;15:1342181. doi: 10.3389/fphar.2024.1342181 38500764 PMC10944884

[94] Wang W, Roberts CJ. Protein aggregation - mechanisms, detection, and control. Int J Pharm. 2018;550(1–2):251–268. doi: 10.1016/j.ijpharm.2018.08.043 30145245

[95] Israel LL, Galstyan A, Cox A, et al. Signature effects of vector-guided systemic nano bioconjugate delivery across blood-brain barrier of normal, Alzheimer’s, and tumor mouse models. ACS Nano. 2022;16(8):11815–11832. doi: 10.1021/acsnano.1c10034 35961653 PMC9413444

[96] Tao B, Du R, Zhang X, et al. Engineering CAR-NK cell derived exosome disguised nano-bombs for enhanced HER2 positive breast cancer brain metastasis therapy. J Control Release. 2023;363:692–706. doi: 10.1016/j.jconrel.2023.10.007 37813124

[97] He W, Li X, Morsch M, et al. Brain-targeted codelivery of Bcl-2/Bcl-xl and Mcl-1 inhibitors by biomimetic nanoparticles for orthotopic glioblastoma therapy. ACS Nano. 2022;16(4):6293–6308. doi: 10.1021/acsnano.2c00320 35353498

[98] Li J, Zhang Z, Zhang B, et al. Transferrin receptor 1 targeted nanomedicine for brain tumor therapy. Biomater Sci. 2023;11(10):3394–3413. doi: 10.1039/D2BM02152H 36847174

[99] Habib S, Singh M. Angiopep-2-modified nanoparticles for brain-directed delivery of therapeutics: a review. Polym (Basel). 2022;14(4):712. doi: 10.3390/polym14040712 PMC8878382 35215625

[100] Jiang S, Li W, Yang J, et al. Cathepsin B-responsive programmed brain targeted delivery system for chemo-immunotherapy combination therapy of glioblastoma. ACS Nano. 2024;18(8):6445–6462. doi: 10.1021/acsnano.3c11958 38358804

[101] Galstyan A, Markman JL, Shatalova ES, et al. Blood-brain barrier permeable nano immunoconjugates induce local immune responses for glioma therapy. Nat Commun. 2019;10(1):3850. doi: 10.1038/s41467-019-11719-3 31462642 PMC6713723

[102] Zhang Z, Li J, Wang Y, et al. Angiopep-2 conjugated biomimetic nano-delivery system loaded with resveratrol for the treatment of methamphetamine addiction. Int J Pharm. 2024;663:124552. doi: 10.1016/j.ijpharm.2024.124552 39111355

[103] Liu X, Cao Z, Wang W, et al. Engineered extracellular vesicle-delivered CRISPR/Cas9 for radiotherapy sensitization of glioblastoma. ACS Nano. 2023;17(17):16432–16447. doi: 10.1021/acsnano.2c12857 37646615 PMC10510715

[104] Zhu Z, Zhai Y, Hao Y, et al. Specific anti-glioma targeted-delivery strategy of engineered small extracellular vesicles dual-functionalised by Angiopep-2 and TAT peptides. J Extracell Vesicles. 2022;11(8):e12255. doi: 10.1002/jev2.12255 35932288 PMC9451528

[105] Israel LL, Braubach O, Galstyan A, et al. A combination of tri-leucine and Angiopep-2 drives a polyanionic polymalic acid nanodrug platform across the blood-brain barrier. ACS Nano. 2019;13(2):1253–1271. doi: 10.1021/acsnano.8b06437 30633492 PMC7641102

[106] Qian K, Bao X, Li Y, et al. Cholinergic neuron targeting nanosystem delivering hybrid peptide for combinatorial mitochondrial therapy in Alzheimer’s disease. ACS Nano. 2022;16(7):11455–11472. doi: 10.1021/acsnano.2c05795 35839463

[107] Spicer CD, Jumeaux C, Gupta B, et al. Peptide and protein nanoparticle conjugates: versatile platforms for biomedical applications. Chem Soc Rev. 2018;47(10):3574–3620. doi: 10.1039/C7CS00877E 29479622 PMC6386136

[108] Aguiar SI, Dias JNR, Andre AS, et al. Highly specific blood-brain barrier transmigrating single-domain antibodies selected by an in vivo phage display screening. Pharmaceutics. 2021;13(10):1598. doi: 10.3390/pharmaceutics13101598 34683891 PMC8540410

[109] Topçu B, Bozdağ Pehlivan S, Akdağ Y, et al. Antibody conjugated nano-enabled drug delivery systems against brain tumors. J Pharm Sci. 2024;113(6):1455–1469. doi: 10.1016/j.xphs.2024.03.017 38555997

[110] Sharma S, Dang S. Nanocarrier-based drug delivery to brain: interventions of surface modification. Curr neuropharmacol. 2023;21(3):517–535. doi: 10.2174/1570159X20666220706121412 35794771 PMC10207924

[111] Ljubimova JY, Ramesh A, Israel LL, et al. Small-sized co-polymers for targeted delivery of multiple imaging and therapeutic agents. Nanomater (Basel). 2021;11(11):2996. doi: 10.3390/nano11112996 PMC8625475 34835760

[112] Alejandra WP, Miriam Irene JP, Fabio Antonio GS, et al. Production of monoclonal antibodies for therapeutic purposes: a review. Int Immunopharmacol. 2023;120:110376. doi: 10.1016/j.intimp.2023.110376 37244118

[113] Li X, Yang Y, Zhao H, et al. Enhanced in vivo blood-brain barrier penetration by circular tau-transferrin receptor bifunctional aptamer for tauopathy therapy. J Am Chem Soc. 2020;142(8):3862–3872. doi: 10.1021/jacs.9b11490 31991082

[114] Su Y, Huang Y, Kou Q, et al. Study on the role of an erythrocyte membrane-coated nanotheranostic system in targeted immune regulation of Alzheimer’s disease. Adv Sci (Weinh). 2023;10(18):e2301361. doi: 10.1002/advs.202301361 37075744 PMC10288270

[115] Guan J, Liu C, Ji C, et al. NIR-II perylene monoimide-based photothermal agent with strengthened donor-acceptor conjugation for deep orthotopic glioblastoma phototheranostics. Small. 2023;19(19):e2300203. doi: 10.1002/smll.202300203 36775955

[116] Wang H, Chao Y, Zhao H, et al. Smart nanomedicine to enable crossing blood-brain barrier delivery of checkpoint blockade antibody for immunotherapy of glioma. ACS Nano. 2022;16(1):664–674. doi: 10.1021/acsnano.1c08120 34978418

[117] Liu D, Cheng Y, Qiao S, et al. Nano-codelivery of temozolomide and siPD-L1 to reprogram the drug-resistant and immunosuppressive microenvironment in orthotopic glioblastoma. ACS Nano. 2022;16(5):7409–7427. doi: 10.1021/acsnano.1c09794 35549164

[118] Duan Q, Liu R, Luo JQ, et al. Virus-inspired glucose and polydopamine (GPDA)-coating as an effective strategy for the construction of brain delivery platforms. Nano Lett. 2024;24(1):402–410. doi: 10.1021/acs.nanolett.3c04175 38153842

[119] Tian Y, Zheng Z, Wang X, et al. Establishment and evaluation of glucose-modified nanocomposite liposomes for the treatment of cerebral malaria. J Nanobiotechnol. 2022;20(1):318. doi: 10.1186/s12951-022-01493-8 PMC9258070 35794597

[120] Yang F, Zhao D, Cheng M, et al. mTOR-mediated immunometabolic reprogramming nanomodulators enable sensitive switching of energy deprivation-induced microglial polarization for Alzheimer’s disease management. ACS Nano. 2023;17(16):15724–15741. doi: 10.1021/acsnano.3c03232 37565731

[121] Du S, Guan Y, Xie A, et al. Extracellular vesicles: a rising star for therapeutics and drug delivery. J Nanobiotechnol. 2023;21(1):231. doi: 10.1186/s12951-023-01973-5 PMC10360328 37475025

[122] Cui J, Wang X, Li J, et al. Immune exosomes loading self-assembled nanomicelles traverse the blood-brain barrier for chemo-immunotherapy against glioblastoma. ACS Nano. 2023;17(2):1464–1484. doi: 10.1021/acsnano.2c10219 36626296

[123] Morad G, Carman CV, Hagedorn EJ, et al. Tumor-derived extracellular vesicles breach the intact blood–brain barrier via transcytosis. ACS Nano. 2019;13(12):13853–13865. doi: 10.1021/acsnano.9b04397 31479239 PMC7169949

[124] Xu F, Wu Y, Yang Q, et al. Engineered extracellular vesicles with SHP2 high expression promote mitophagy for Alzheimer’s disease treatment. Adv Mater. 2022;34(49):e2207107. doi: 10.1002/adma.202207107 36193769

[125] Shan S, Chen J, Sun Y, et al. Functionalized macrophage exosomes with panobinostat and PPM1D-siRNA for diffuse intrinsic pontine gliomas therapy. Adv Sci (Weinh). 2022;9(21):e2200353. doi: 10.1002/advs.202200353 35585670 PMC9313473

[126] Niu W, Xiao Q, Wang X, et al. A biomimetic drug delivery system by integrating grapefruit extracellular vesicles and doxorubicin-loaded heparin-based nanoparticles for glioma therapy. Nano Lett. 2021;21(3):1484–1492. doi: 10.1021/acs.nanolett.0c04753 33475372

[127] Kim J, Zhu Y, Chen S, et al. Anti-glioma effect of ginseng-derived exosomes-like nanoparticles by active blood-brain-barrier penetration and tumor microenvironment modulation. J Nanobiotechnol. 2023;21(1):253. doi: 10.1186/s12951-023-02006-x PMC10401762 37542285

[128] Ma X, Kuang L, Yin Y, et al. Tumor-antigen activated dendritic cell membrane-coated biomimetic nanoparticles with orchestrating immune responses promote therapeutic efficacy against glioma. ACS Nano. 2023;17(3):2341–2355. doi: 10.1021/acsnano.2c09033 36688797

[129] Sun Y, Kong J, Ge X, et al. An antisense oligonucleotide-loaded blood-brain barrier penetrable nanoparticle mediating recruitment of endogenous neural stem cells for the treatment of Parkinson’s disease. ACS Nano. 2023;17(5):4414–4432. doi: 10.1021/acsnano.2c09752 36688425

[130] Jia X, Wang L, Feng X, et al. Cell membrane-coated oncolytic adenovirus for targeted treatment of glioblastoma. Nano Lett. 2023;23(23):11120–11128. doi: 10.1021/acs.nanolett.3c03516 38032110

[131] Peng Y, Zhan M, Karpus A, et al. Brain delivery of biomimetic phosphorus dendrimer/antibody nanocomplexes for enhanced glioma immunotherapy via immune modulation of T cells and natural killer cells. ACS Nano. 2024;18(14):10142–10155. doi: 10.1021/acsnano.3c13088 38526307

[132] Yin T, Fan Q, Hu F, et al. Engineered macrophage-membrane-coated nanoparticles with enhanced PD-1 expression induce immunomodulation for a synergistic and targeted antiglioblastoma activity. Nano Lett. 2022;22(16):6606–6614. doi: 10.1021/acs.nanolett.2c01863 35948420

[133] Xiao T, He M, Xu F, et al. Macrophage membrane-camouflaged responsive polymer nanogels enable magnetic resonance imaging-guided chemotherapy/chemodynamic therapy of orthotopic glioma. ACS Nano. 2021;15(12):20377–20390. doi: 10.1021/acsnano.1c08689 34860014

[134] He W, Mei Q, Li J, et al. Preferential targeting cerebral ischemic lesions with cancer cell-inspired nanovehicle for ischemic stroke treatment. Nano Lett. 2021;21(7):3033–3043. doi: 10.1021/acs.nanolett.1c00231 33755480

[135] Li J, Wei Y, Zhang C, et al. Cell-membrane-coated nanoparticles for targeted drug delivery to the brain for the treatment of neurological diseases. Pharmaceutics. 2023;15(2):621. doi: 10.3390/pharmaceutics15020621 36839943 PMC9960717

[136] Ma Y, Yi J, Ruan J, et al. Engineered cell membrane-coated nanoparticles: new strategies in glioma targeted therapy and immune modulation. Adv Healthc Mater. 2024;13(20):e2400514. doi: 10.1002/adhm.202400514 38652681

[137] Yang J, Wang P, Jiang X, et al. A nanotherapy of octanoic acid ameliorates cardiac arrest/cardiopulmonary resuscitation-induced brain injury via RVG29- and neutrophil membrane-mediated injury relay targeting. ACS Nano. 2023;17(4):3528–3548. doi: 10.1021/acsnano.2c09931 36758159

[138] Fan L, Jin L, Tang T, et al. Neutrophil-like pH-responsive pro-efferocytic nanoparticles improve neurological recovery by promoting erythrophagocytosis after intracerebral hemorrhage. Theranostics. 2024;14(1):283–303. doi: 10.7150/thno.90370 38164152 PMC10750197

[139] Cao Z, Liu X, Zhang W, et al. Biomimetic macrophage membrane-camouflaged nanoparticles induce ferroptosis by promoting mitochondrial damage in glioblastoma. ACS Nano. 2023;17(23):23746–23760. doi: 10.1021/acsnano.3c07555 37991252 PMC10722604

[140] Lu J, Ding J, Chu B, et al. Inactive trojan bacteria as safe drug delivery vehicles crossing the blood-brain barrier. Nano Lett. 2023;23(10):4326–4333. doi: 10.1021/acs.nanolett.3c00563 37130058

[141] Pan J, Wang Z, Huang X, et al. Bacteria-derived outer-membrane vesicles hitchhike neutrophils to enhance ischemic stroke therapy. Adv Mater. 2023;35(38):e2301779. doi: 10.1002/adma.202301779 37358255

[142] Chen H, Zhang S, Fang Q, et al. Biomimetic nanosonosensitizers combined with noninvasive ultrasound actuation to reverse drug resistance and sonodynamic-enhanced chemotherapy against orthotopic glioblastoma. ACS Nano. 2023;17(1):421–436. doi: 10.1021/acsnano.2c08861 36573683

[143] Padilla F, Brenner J, Prada F, et al. Theranostics in the vasculature: bioeffects of ultrasound and microbubbles to induce vascular shutdown. Theranostics. 2023;13(12):4079–4101. doi: 10.7150/thno.70372 37554276 PMC10405856

[144] Kwak G, Grewal A, Slika H, et al. Brain nucleic acid delivery and genome editing via focused ultrasound-mediated blood-brain barrier opening and long-circulating nanoparticles. ACS Nano. 2024;18(35):24139–24153. doi: 10.1021/acsnano.4c05270 39172436 PMC11792178

[145] Kong W, Li X, Guo X, et al. Ultrasound-assisted CRISPRi-exosome for epigenetic modification of alpha-synuclein gene in a mouse model of Parkinson’s disease. ACS Nano. 2024;18(11):7837–7851. doi: 10.1021/acsnano.3c05864 38437635

[146] Wang J, Xie L, Shi Y, et al. Early detection and reversal of cell apoptosis induced by focused ultrasound-mediated blood-brain barrier opening. ACS Nano. 2021;15(9):14509–14521. doi: 10.1021/acsnano.1c04029 34405679

[147] Shirvalilou S, Khoei S, Khoee S, et al. Enhancement radiation-induced apoptosis in C6 glioma tumor-bearing rats via pH-responsive magnetic graphene oxide nanocarrier. J Photochem Photobiol B. 2020;205:111827. doi: 10.1016/j.jphotobiol.2020.111827 32120183

[148] Chen J, Yuan M, Madison CA, et al. Blood-brain barrier crossing using magnetic stimulated nanoparticles. J Control Release. 2022;345:557–571. doi: 10.1016/j.jconrel.2022.03.007 35276300

[149] Li B, Chen X, Qiu W, et al. Synchronous disintegration of ferroptosis defense axis via engineered exosome‐conjugated magnetic nanoparticles for glioblastoma therapy. Adv Sci. 2022;9(17). doi: 10.1002/advs.202105451 PMC9189685 35508804

[150] Gupta R, Chauhan A, Kaur T, et al. Transmigration of magnetite nanoparticles across the blood-brain barrier in a rodent model: influence of external and alternating magnetic fields. Nanoscale. 2022;14(47):17589–17606. doi: 10.1039/D2NR02210A 36409463

[151] Kaushik A, Yndart A, Atluri V, et al. Magnetically guided non-invasive CRISPR-Cas9/gRNA delivery across blood-brain barrier to eradicate latent HIV-1 infection. Sci Rep. 2019;9(1):3928. doi: 10.1038/s41598-019-40222-4 30850620 PMC6408460

[152] Li X, Vemireddy V, Cai Q, et al. Reversibly modulating the blood-brain barrier by laser stimulation of molecular-targeted nanoparticles. Nano Lett. 2021;21(22):9805–9815. doi: 10.1021/acs.nanolett.1c02996 34516144 PMC8616836

[153] Du C, Feng W, Dai X, et al. Cu2+‐chelatable and ROS‐scavenging MXenzyme as NIR‐II‐triggered blood–brain barrier‐crossing nanocatalyst against Alzheimer’s disease. Small. 2022;18(39). doi: 10.1002/smll.202203031 36008124

[154] Zhang Y, Xi K, Zhang Y, et al. Blood-brain barrier penetrating nanovehicles for interfering with mitochondrial electron flow in glioblastoma. ACS Nano. 2024;18(13):9511–9524. doi: 10.1021/acsnano.3c12434 38499440

[155] Liu Y, Zou Y, Feng C, et al. Charge conversional biomimetic nanocomplexes as a multifunctional platform for boosting orthotopic glioblastoma RNAi therapy. Nano Lett. 2020;20(3):1637–1646. doi: 10.1021/acs.nanolett.9b04683 32013452

[156] Fan Q, Kuang L, Wang B, et al. Multiple synergistic effects of the microglia membrane-bionic nanoplatform on mediate tumor microenvironment remodeling to amplify glioblastoma immunotherapy. ACS Nano. 2024;18(22):14469–14486. doi: 10.1021/acsnano.4c01253 38770948

[157] Su L, Zhu K, Ge X, et al. X-ray activated nanoprodrug for visualization of cortical microvascular alterations and NIR-II image-guided chemo-radiotherapy of glioblastoma. Nano Lett. 2024;24(12):3727–3736. doi: 10.1021/acs.nanolett.4c00223 38498766

[158] Ma M, Gao N, Li X, et al. A biocompatible second near-infrared nanozyme for spatiotemporal and non-invasive attenuation of amyloid deposition through scalp and skull. ACS Nano. 2020;14(8):9894–9903. doi: 10.1021/acsnano.0c02733 32806077

[159] Xu K, Huang P, Wu Y, et al. Engineered selenium/human serum albumin nanoparticles for efficient targeted treatment of Parkinson’s disease via oral gavage. ACS Nano. 2023;17(20):19961–19980. doi: 10.1021/acsnano.3c05011 37807265 PMC10604087

[160] Marino A, Battaglini M, Desii A, et al. Liposomes loaded with polyphenol-rich grape pomace extracts protect from neurodegeneration in a rotenone-based in vitro model of Parkinson’s disease. Biomater Sci. 2021;9(24):8171–8188. doi: 10.1039/D1BM01202A 34617936

[161] Li B, Bai Y, Yion C, et al. Single-atom nanocatalytic therapy for suppression of neuroinflammation by inducing autophagy of abnormal mitochondria. ACS Nano. 2023;17(8):7511–7529. doi: 10.1021/acsnano.2c12614 37018124

[162] Hong W, Zhang Z, Liu L, et al. Brain-targeted delivery of PEGylated nano-bacitracin a against penicillin-sensitive and -resistant pneumococcal meningitis: formulated with RVG(29) and Pluronic((R)) P85 unimers. Drug Deliv. 2018;25(1):1886–1897. doi: 10.1080/10717544.2018.1486473 30404541 PMC6225518

[163] Perez-Arizti JA, Ventura-Gallegos JL, Galvan Juarez RE, et al. Titanium dioxide nanoparticles promote oxidative stress, autophagy and reduce NLRP3 in primary rat astrocytes. Chem Biol Interact. 2020;317:108966. doi: 10.1016/j.cbi.2020.108966 32004531

[164] Huang T, Guo Y, Wang Z, et al. Biomimetic dual-target theranostic nanovaccine enables magnetic resonance imaging and chemo/chemodynamic/immune therapy of glioma. ACS Appl Mater Interface. 2024;16(21):27187–27201. doi: 10.1021/acsami.4c05831 38747985

[165] Reichel D, Sagong B, Teh J, et al. Near infrared fluorescent nanoplatform for targeted intraoperative resection and chemotherapeutic treatment of glioblastoma. ACS Nano. 2020;14(7):8392–8408. doi: 10.1021/acsnano.0c02509 32551496 PMC7438253

[166] Jiang S, Cai G, Yang Z, et al. Biomimetic nanovesicles as a dual gene delivery system for the synergistic gene therapy of Alzheimer’s disease. ACS Nano. 2024;18(18):11753–11768. doi: 10.1021/acsnano.3c13150 38649866

[167] Huang D, Wang Q, Cao Y, et al. Multiscale NIR-II imaging-guided brain-targeted drug delivery using engineered cell membrane nanoformulation for Alzheimer’s disease therapy. ACS Nano. 2023;17(5):5033–5046. doi: 10.1021/acsnano.2c12840 36867454

[168] Han Y, Yi H, Wang Y, et al. Ultrathin zinc selenide nanoplatelets boosting photoacoustic imaging of in situ copper exchange in Alzheimer’s disease mice. ACS Nano. 2022;16(11):19053–19066. doi: 10.1021/acsnano.2c08094 36349982

[169] Garcia-Pardo J, Novio F, Nador F, et al. Bioinspired theranostic coordination polymer nanoparticles for intranasal dopamine replacement in Parkinson’s disease. ACS Nano. 2021;15(5):8592–8609. doi: 10.1021/acsnano.1c00453 33885286 PMC8558863

[170] Chen HJ, Qin Y, Wang ZG, et al. An activatable and reversible virus-mimicking NIR-II nanoprobe for monitoring the progression of viral encephalitis. Angew Chem Int Ed Engl. 2022;61(39):e202210285. doi: 10.1002/anie.202210285 35965257

[171] Shi R, Lv R, Dong Z, et al. Magnetically-targetable outer-membrane vesicles for sonodynamic eradication of antibiotic-tolerant bacteria in bacterial meningitis. Biomaterials. 2023;302:122320. doi: 10.1016/j.biomaterials.2023.122320 37738742

[172] Wang X, Zhang W. The Janus of protein corona on nanoparticles for tumor targeting, immunotherapy and diagnosis. J Control Release. 2022;345:832–850. doi: 10.1016/j.jconrel.2022.03.056 35367478

[173] Chen ZA, Wu CH, Wu SH, et al. Receptor ligand-free mesoporous silica nanoparticles: a streamlined strategy for targeted drug delivery across the blood-brain barrier. ACS Nano. 2024;18(20):12716–12736. doi: 10.1021/acsnano.3c08993 38718220 PMC11112986

[174] Li J, Long Q, Ding H, et al. Progress in the treatment of central nervous system diseases based on nanosized traditional Chinese medicine. Adv Sci (Weinh). 2024;11(16):e2308677. doi: 10.1002/advs.202308677 38419366 PMC11040388

[175] Wei D, Yang H, Zhang Y, et al. Nano-traditional Chinese medicine: a promising strategy and its recent advances. J Mater Chem B. 2022;10(16):2973–2994. doi: 10.1039/D2TB00225F 35380567

